# Increased mosquito abundance and species richness in Connecticut, United States 2001–2019

**DOI:** 10.1038/s41598-020-76231-x

**Published:** 2020-11-06

**Authors:** Tanya A. Petruff, Joseph R. McMillan, John J. Shepard, Theodore G. Andreadis, Philip M. Armstrong

**Affiliations:** Center for Vector Biology and Zoonotic Diseases, Environmental Sciences, The Connecticut Agricultural Experiment Station, 123 Huntington Street, New Haven, CT 06511 USA

**Keywords:** Ecology, Zoology

## Abstract

Historical declines in multiple insect taxa have been documented across the globe in relation to landscape-level changes in land use and climate. However, declines have either not been universally observed in all regions or examined for all species. Because mosquitoes are insects of public health importance, we analyzed a longitudinal mosquito surveillance data set from Connecticut (CT), United States (U.S.) from 2001 to 2019 to identify changes in mosquito community composition over time. We first analyzed annual site-level collections and metrics of mosquito community composition with generalized linear/additive mixed effects models; we also examined annual species-level collections using the same tools. We then examined correlations between statewide collections and weather variables as well as site-level collections and land cover classifications. We found evidence that the average trap night collection of mosquitoes has increased by ~ 60% and statewide species richness has increased by ~ 10% since 2001. Total species richness was highest in the southern portion of CT, likely due to the northward range expansion of multiple species within the *Aedes*, *Anopheles, Culex, *and* Psorophora* genera. How the expansion of mosquito populations in the northeast U.S. will alter mosquito-borne pathogen transmission in the region will require further investigation.

## Introduction

Recent publications analyzing longitudinal data of insect populations have indicated an overall decline in insect diversity (predominately species richness) and abundance in North America and Europe in the last half century^1,2^. These declines are driven by a variety of factors including changes in landscape such as increased urbanization and deforestation^1^ and weather patterns, i.e., global climate change. In addition, parasitic diseases and the widespread use of systemic insecticides, such as neonicotinoids, have been implicated in the decline of pollinating insects, such as bees^3^. These studies have raised public alarm given that insects are critical members of biological communities and ecosystem processes. However, declines across all insect communities have not been universally observed. Moth biomass in the United Kingdom has increased since the 1960s^4^, freshwater aquatic insect diversity has increased in countries that enacted water pollution regulations^2^, and historical increases in mosquito species richness and abundance across the U.S. is linked to insecticide usage restrictions such as banning the use of DDT^5^. Overall, anthropogenic-driven environmental changes are having broad-scale impacts on insect communities, which will have important environmental, economic, and public health implications in the present and future.

Mosquitoes are an important group of insects to monitor in the context of landscape and climate change due to their ability to vector human and animal pathogens as well as their impact on human enjoyment of the environment. Additionally, because mosquitoes are important to public health, mosquito surveillance data sets offer many advantages for understanding changes in insect populations: most mosquito surveillance techniques are standardized^6^, dichotomous keys and identification guides exist for many regions of the world^7^, and their bipartite lifecycle (larvae develop in aquatic habitats and adults are terrestrial) make them sensitive indicators to fine-scale changes in habitat and climate^8^. However, there are two major limitations to investigating longitudinal dynamics of mosquito populations and their subsequent communities. The first, and most important, limitation to understanding long-term dynamics of mosquito populations is a lack of long-term data. Some municipal and regional level mosquito control districts in the U.S. have data sets dating back decades and maintain long term surveillance sites; however, these data are the exception and the majority of mosquito field studies are short (e.g., 1–3 years) in duration. The second limitation to understanding longitudinal trends in mosquito population is the central focus on either single species or community dynamics in the context of disease transmission^9^. A single species (or pathogen) surveillance approach has increased the prevalence of long-term data on West Nile virus (WNV) transmission in the U.S., especially in regards to the *Culex pipiens* (Linnaeus) species complex^10^ and the invasion dynamics of *Aedes albopictus* (Skuse)^11^, yet these data sets do little to improve the understanding of mosquito species community dynamics in general. This lack of general mosquito community ecology knowledge is a critical gap in our ability to control mosquito populations and forecast disease (re)emergence^12^.

There is some research that explores how mosquito communities are changing due to landscape-level dynamics. Previous studies on mosquito community structure have shown reductions in mosquito species richness and increases in the dominance of vectors of public health importance along forested to urban gradients^13^, simplification of mosquito community structure in homogenized landscapes^14^, changes in human risk of mosquito-borne pathogens linked to habitat disturbances^15,16^, and in some instances, increased mosquito species richness was associated with increased pathogen prevalence^17,18^. Globalization has also led to the introduction and establishment of mosquito species (and mosquito-borne pathogens) outside their evolutionary origins^19,20^. Historically, interventions such as mosquito control efforts to suppress malaria and yellow fever had a large and negative impact on mosquito abundance and community richness^5^; however, such broad-scale campaigns are no longer operable in many regions of the world and numerous mosquito populations have likely rebounded throughout much of their ranges.

In response to global climate change (more specifically global warming), many mosquito species and their associated pathogens are predicted to expand their geographical distributions with warming temperatures^20,21^. In the U.S., species such as *Culex coronator* (Dyar and Knab) and *Culex erraticus* (Dyar and Knab) in Central America and the southern U.S. have been documented in northern latitudes outside of these species’ historical distributions^22,23^. Other studies have shown changes in mosquito species distributions linked with temperature changes along altitudinal gradients^24,25^ (though these changes in altitudinal gradients could also be driven by changes in land use) and projected range expansions under various climate change scenarios^21^. Overall, identifying species expansions strictly due to warming temperatures is difficult to assess as changes in climate are often coupled with factors such as land use changes and human behavior that feedback into climate change.

In respect to mosquito species communities in the northeast region of the U.S., Andreadis et al. (2005) published an identification guide to mosquitoes in Connecticut (CT) which at the time documented forty-nine mosquito species in the state^26^. Prior to publication of the guide, the 0 °C winter isotherm was proposed as a limiting gradient to the expansion of *Aedes albopictus* and other more southern species incapable of adult diapause or producing eggs resistant to winter conditions^27^. However, *Ae. albopictus* is currently established in the northeast^28^, and mosquito species richness has increased annually throughout CT from 2001 to 2018^29^. To better understand changes in mosquito species populations in the northeast U.S., our study objectives were to identify spatial and temporal trends in metrics of mosquito community composition that would indicate growth or decline of mosquito populations among sites and species. Our site-level regression analyses focused on total collections, species richness, species evenness, and the proportion of single-species detections; we also examined correlations between these metrics and land cover and seasonal weather variables. Our species-level regression analyses focused on total collections and the prevalence of single-site detections; we also provide an inventory of newly documented, recently established, and possibly declining mosquito species in the Northeast, and discuss species of emerging public health importance in the region.

## Results

### Summary statistics

**Figure 1:**
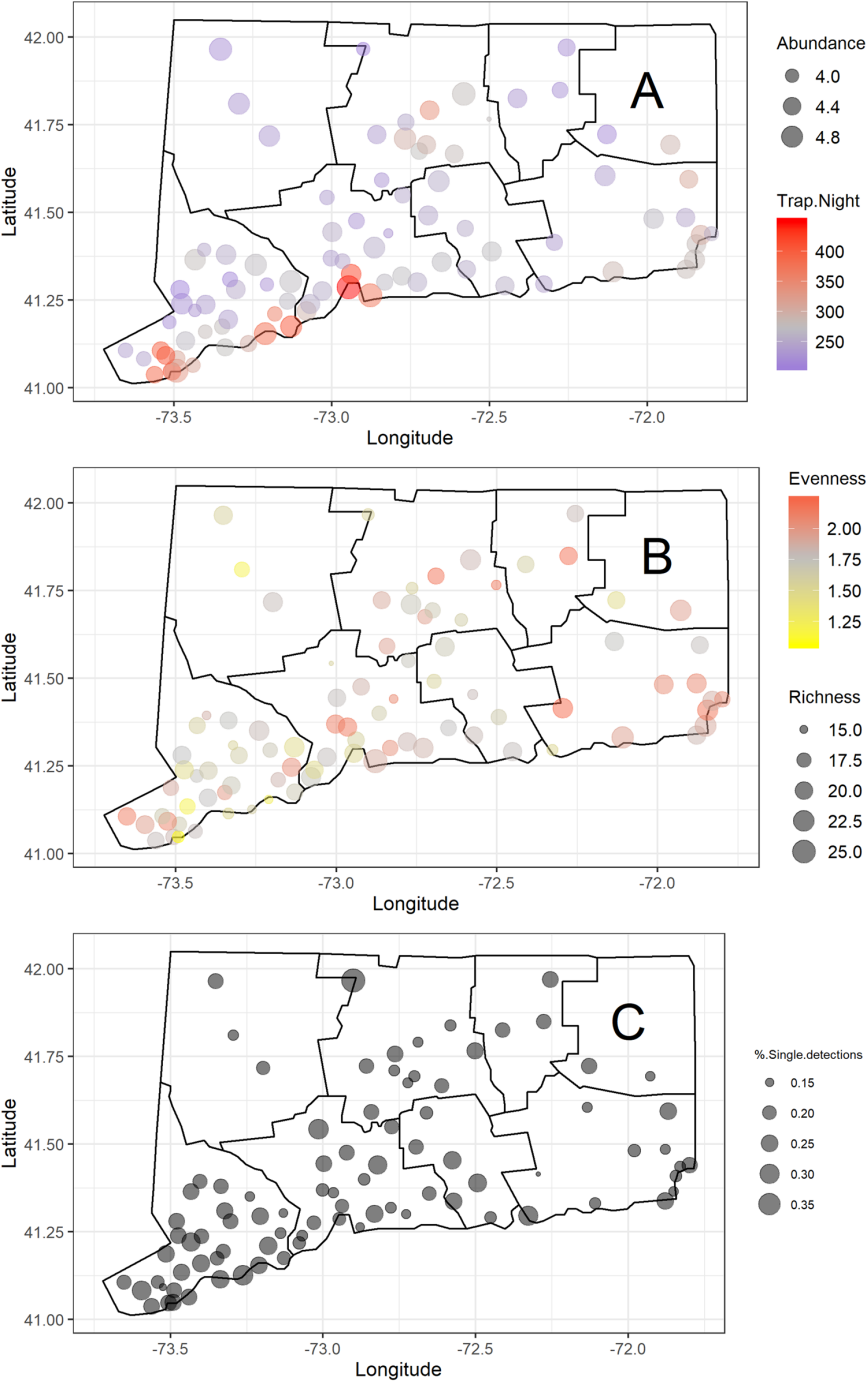
Maps of total mosquito abundance (log_10_ transformed) (**A**), total number of trap nights (**A**), average annual mosquito species richness (**B**), average annual mosquito species evenness (**B**), and average annual prevalence of single species detections (**C**) across 87 mosquito surveillance sites throughout Connecticut, U.S. sampled with ground level CDC CO_2_-baited light traps from 2001 to 2019. (**A**) Point sizes represent abundance while colors represent trap-nights; (**B**) point sizes represent species richness while colors represent species evenness; (**C**) point sizes represent prevalence of single species detections. (**A**–**C**) Solid black lines represent county political boundaries. The figure was created in R V 3.6.3 using the following packages: *ggplot2* and *maps*.

To date, The Connecticut Agricultural Experiment Station (CAES) has collected and tested 4,602,240 female mosquitoes comprised of 47 species in 8 genera. Approximately 98% of these collections were obtained from 92 trapping sites in 73 towns throughout the state, while the remainder of collections were from an additional 365 supplemental sites sampled between 1996 and 2007. Eighty-eight percent of collections come from CDC Light Traps, CDC Gravid Traps and Biogents BG Sentinel Traps (beginning in 2012). There have been several other collection methods used throughout the years that account for 11.6% of the mosquitoes collected (S. Table 1). Overall, there was considerable variation in mosquito abundance, surveillance effort, species richness/evenness, and the proportion of single species detections across CT (Fig. 1). One clear trend was that surveillance effort was greatest in CT’s human population centers (predominately CT’s southwestern and central counties) where WNV is commonly detected and along the CT-Rhode Island border where EEEV is most commonly detected (Fig. 1A). Another noticeable visual trend was that species evenness tends to be higher in the eastern portion of CT (Fig. 1B).

### Objective 1: annual collections of mosquito populations among sites

Our first objective was to identify spatial and temporal linear and nonlinear trends in mosquito abundance among sites. We also examined coarse-scale correlations between statewide (i.e., annual) and site-wide abundance and weather and land classification variables. All regression results and tables are provided as supporting information in *Supporting Information: Regression Tables*.

#### Mosquito abundance

##### Temporal regressions

**Figure 2:**
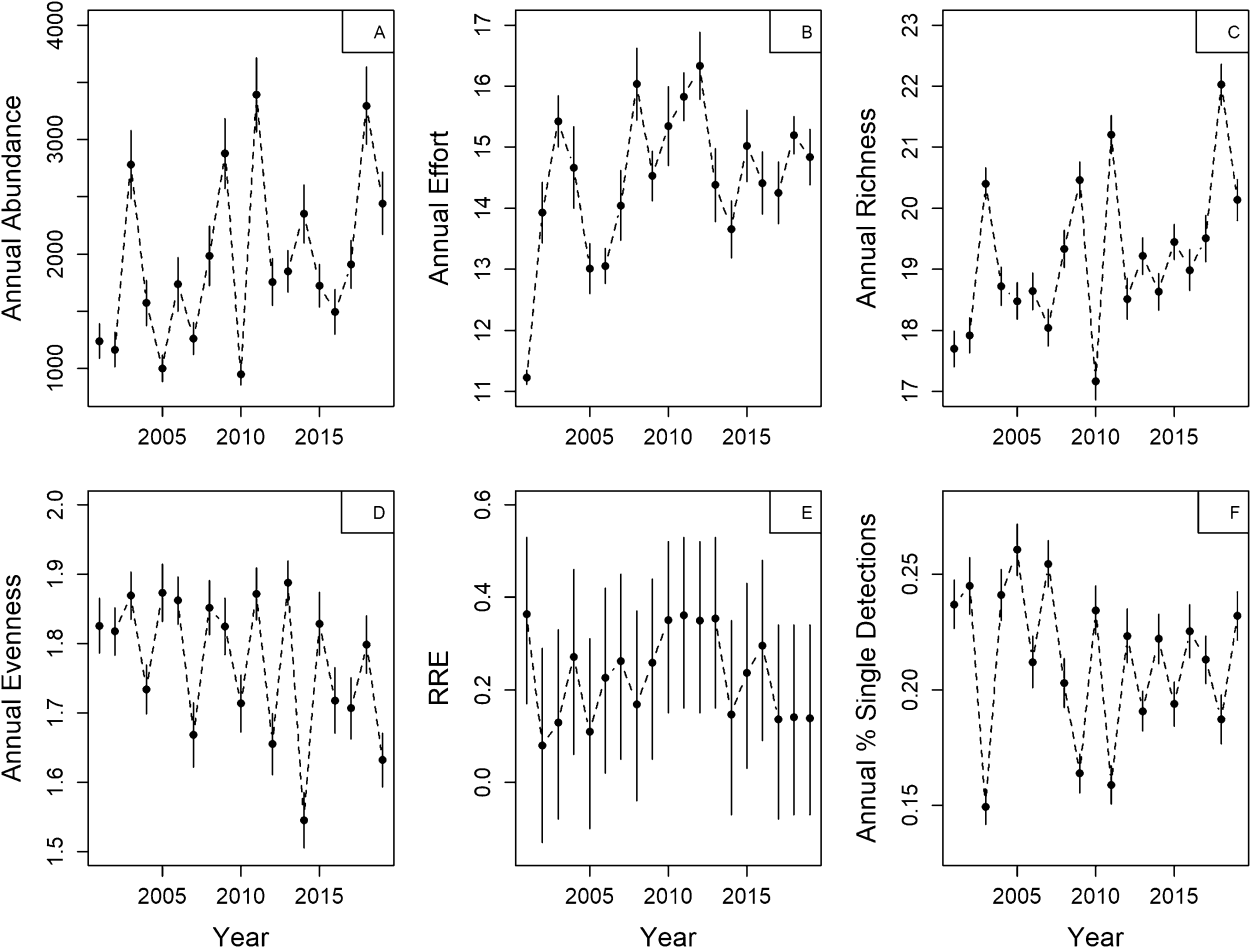
Average annual mosquito abundance (**A**), number of trap nights (**B**), mosquito species richness (**C**), mosquito species evenness (**D**), the annual correlation between mosquito species richness and evenness (**E**), and the prevalence of single mosquito species detections (**F**) across 87 mosquito surveillance sites throughout Connecticut, U.S. sampled with ground level CDC CO_2_-baited light traps from 2001 – 2019. For (**A**)–(**D**) and (**F**), points represent the average across all sites, solid lines represent the standard error of the average, and dashed lines are added to aid interpreting each plot as a time series. For (**E**), points represent the average across all sites while solid lines represent the 95% CI of the correlation point estimate. The figure was created in R V 3.6.3 using base functions.

After accounting for trapping effort, regression parameters estimating the relationship between site-level mosquito abundance and year of collection were positive using generalized linear mixed effects models (GLMMs) (“Year”—Estimate 0.03, t-value 9.11) and generalized additive mixed effects models (GAMMs) (“Year”—Est. 0.77, t-value 2.7, p = 0.007), suggesting that site-level mosquito abundance has increased in CT since 2001 (Fig. 2A,B): this trend resulted in a predicted 60% increase in annual abundance from 2001 to 2019. While these regressions identified possible increasing trends in site-level abundance, they provided an overall poor-fit to the data: AIC scores from fixed effect GLMMs were higher than random effects-only models (ΔAIC 415.1). This poor model fit may be in part driven by directly modeling Year as a fixed continuous effect; Year as a random categorical effect may better capture variation in mosquito collections^30^. Despite large differences in AIC scores between fixed and random effects-only models, we detected a pattern of increasing intercept values when examining “Year” as a random effect (S. Fig. 1), providing further evidence of an increasing temporal trend in site-level mosquito abundance.

##### Spatial regressions

After accounting for trapping effort, regression parameters estimating the relationship between site-level mosquito abundance and latitude/longitude were positive using a GLMM (“Latitude (centered)”—Est. 0.49, t-value 5.48; Longitude (centered)”—Est. 0.20, t-value 4.78), indicating that mosquito abundance tends to increase on a south to north and west to east gradient (which reflects the overall transition in land cover from developed to forested in CT). The best fitting fixed effect GAMM included Longitude by Latitude smoothing terms, which also predicted positive relationships between abundance and site coordinates (Smoothing term 1: Est. 0.24, p = 0.06; Smoothing term 2: Est. 0.05, p = 0.67). GAMM predictions of site-level mosquito abundance were considerably more complex than GLMM predictions, yet still supported the overall trend of increasing abundance from south to north and west to east (S. Fig. 2). Overall, the fixed effect GLMMs provided an extremely poor fit to the data compared to random effects-only GLMMs (Latitude—ΔAIC 1092.7; Longitude—ΔAIC 1099.8). These poor model fits may be in part driven by directly modeling coordinate (i.e., site) as a fixed continuous effect: GAMM predictions that account for nonlinear relationships between abundance and spatial location may provide a more appropriate fit to the data while site as a categorical random effect in the GLMMs may better capture variation in mosquito collections^30^.

##### Weather correlations

When comparing statewide annual mosquito abundance to weather variables, we found no correlations between summer temperatures, spring temperatures or precipitation. This was despite detecting a slight annual increase in temperatures across all three seasons examined (average daily temperature GLMM Est., Season/Summer: 0.05 °C, Prior Spring: 0.02 °C, Prior Winter: 0.07 °C) and a slight annual decline in within season and prior spring precipitation (total precipitation GLMM Est., Season/Summer: − 4.23 mm, Prior Spring: − 3.38 mm; Prior Winter: 2.22 mm) in CT since 2001. However, we did find a positive correlation between total summer precipitation and annual statewide mosquito abundance (r = 0.50, CI 0.07–0.78).

##### Land cover correlations

When comparing total site-wide abundance to land cover classifications, we found positive correlations between percent land cover categorized as barren (r = 0.22, CI 0.01–0.41), forested wetland (r = 0.34, 0.14–0.52), and non-forested wetland (r = 0.21, 0.004–0.41). We also found a negative association in total site-level abundance and percent land cover categorized as grass (r = − 0.35, − 0.52 to − 0.15).

#### Species richness

##### Temporal regressions

**Figure 3:**
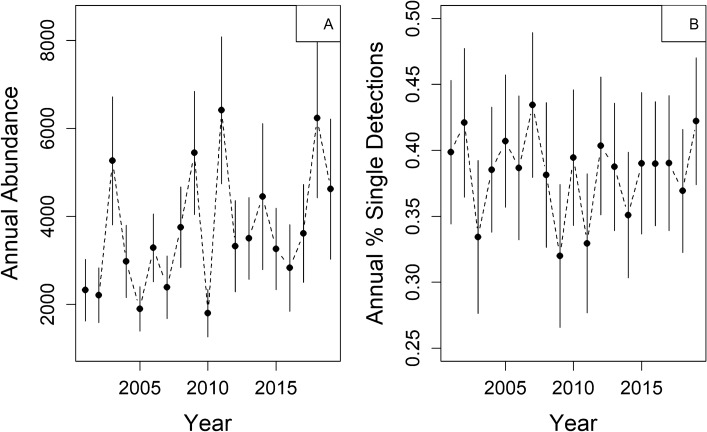
Average annual mosquito abundance (**A**) and the prevalence of single site detections (**B**) across 46 commonly captured mosquito species in Connecticut, U.S. All individuals were collected across 87 sites sampled with ground-level CDC CO_2_-baited light traps from 2001 to 2019. Points represent the average across all species, solid lines represent the standard error of the average, and dashed lines are added to aid interpreting each plot as a time series. The figure was created in R V 3.6.3 using base functions.

After accounting for trapping effort, regression parameters estimating the relationship between site-level species richness and year of collection were positive using both GLMMs (“Year (centered)”—Est. 0.10, t-value 9.46) and GAMMs (“Year”—Est. 1.78, t-value 1.93, p = 0.05) (Fig. 2C): this trend resulted in a predicted 10% increase in site-level species richness from 2001 to 2019. Overall, fixed effects GLMMs of species richness provided an overall poor fit to the data when compared to a random effects-only model (ΔAIC 319.37). However, we did observe a pattern of increasing intercept values when examining “Year” as a random effect (S. Fig. 3), further indicating that mosquito species richness has annually increased across sites in CT since 2001.

##### Spatial regressions

**Figure 4:**
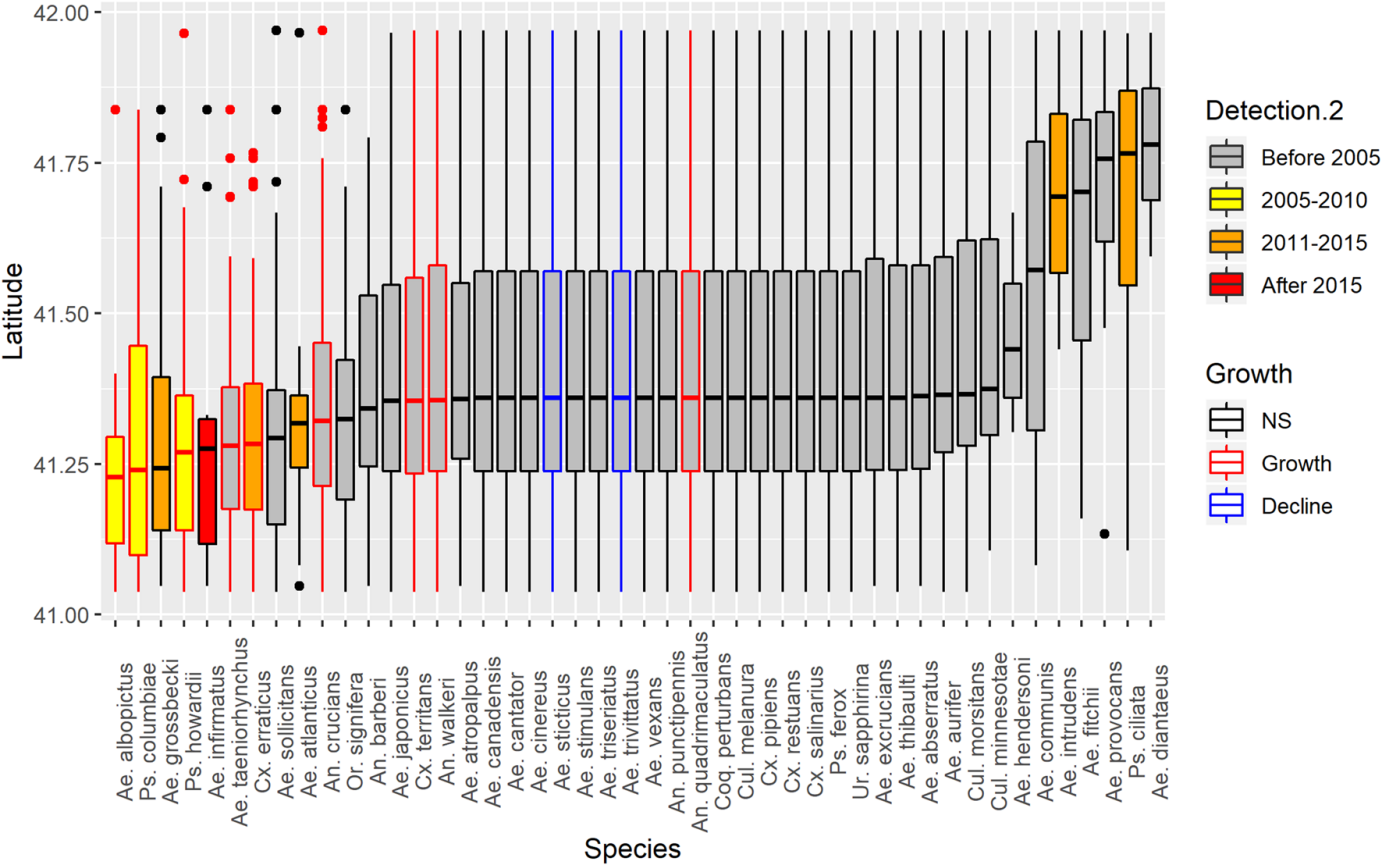
The latitudinal distribution of forty-six mosquito species collected in light traps across 87 surveillance locations in Connecticut, U.S. sampled with ground-level CO_2_-baited light traps from 2001 to 2019. Species are ordered by their average location of detection across all light trap collections in the CAES database. Fill colors represent the time period of first detection during standardized surveillance; border colors indicate statistical evidence of growth or decline in collections throughout CT; *NS* not significant. Species listed in Table 1 which are not listed here include *Aedes flavescens* and *Aedes spencerii* as there are only one collection of each species across all trapping effort. The figure was created in R V 3.6.3 using the *ggplot2* package.

Similar to models of site-level mosquito abundance, GLMMs of species richness by coordinate predicted positive relationships (Latitude (centered): Est. 0.63, t-value = 2.11; Longitude (centered): Est. 1.26, t-value = 9.34), indicating the species richness tends to increase along a south to north, west to east gradient. The best fitting GAMM included Longitude by Latitude smoothing terms, which also predicted positive relationships between species richness and site coordinate (Smoothing term 1: Est. 1.45, p = 0.0001; Smoothing term 2: Est. 0.70, p = 0.05). The GAMM further predicted a complex relationship of species richness among sites, yet overall predicted richness was lowest in the southwest/central portions of CT (areas of greatest development) and highest along coastal/eastern portions of CT (areas of non-forested and forested wetlands) (S. Fig. 4). The fixed effect GLMMs provided very poor fits to the data compared with random effects-only models (Latitude: ΔAIC 953.01; Longitude: ΔAIC 871.93; see the above results for *Site-level collections: spatial regressions* for possible reasons for these poor fits).

##### Weather correlations

We found no correlations of note between mosquito species richness and seasonal temperatures and precipitation.

##### Land cover correlations

Positive correlations of note for site-level species richness included: coniferous forest (r = 0.25, 0.04–0.43), deciduous forest (r = 0.56, 0.40–0.69), and forested wetland (r = 0.43, 0.23–0.58). Negative correlations included: barren (r = − 0.30, − 0.48 to − 0.10), developed (r = − 0.66, − 0.77 to − 0.53), grass (r = − 0.24, − 0.43 to − 0.03), and open water (r = − 0.31, − 0.49 to − 0.11).

#### Species evenness

##### Temporal regressions

**Figure 5:**
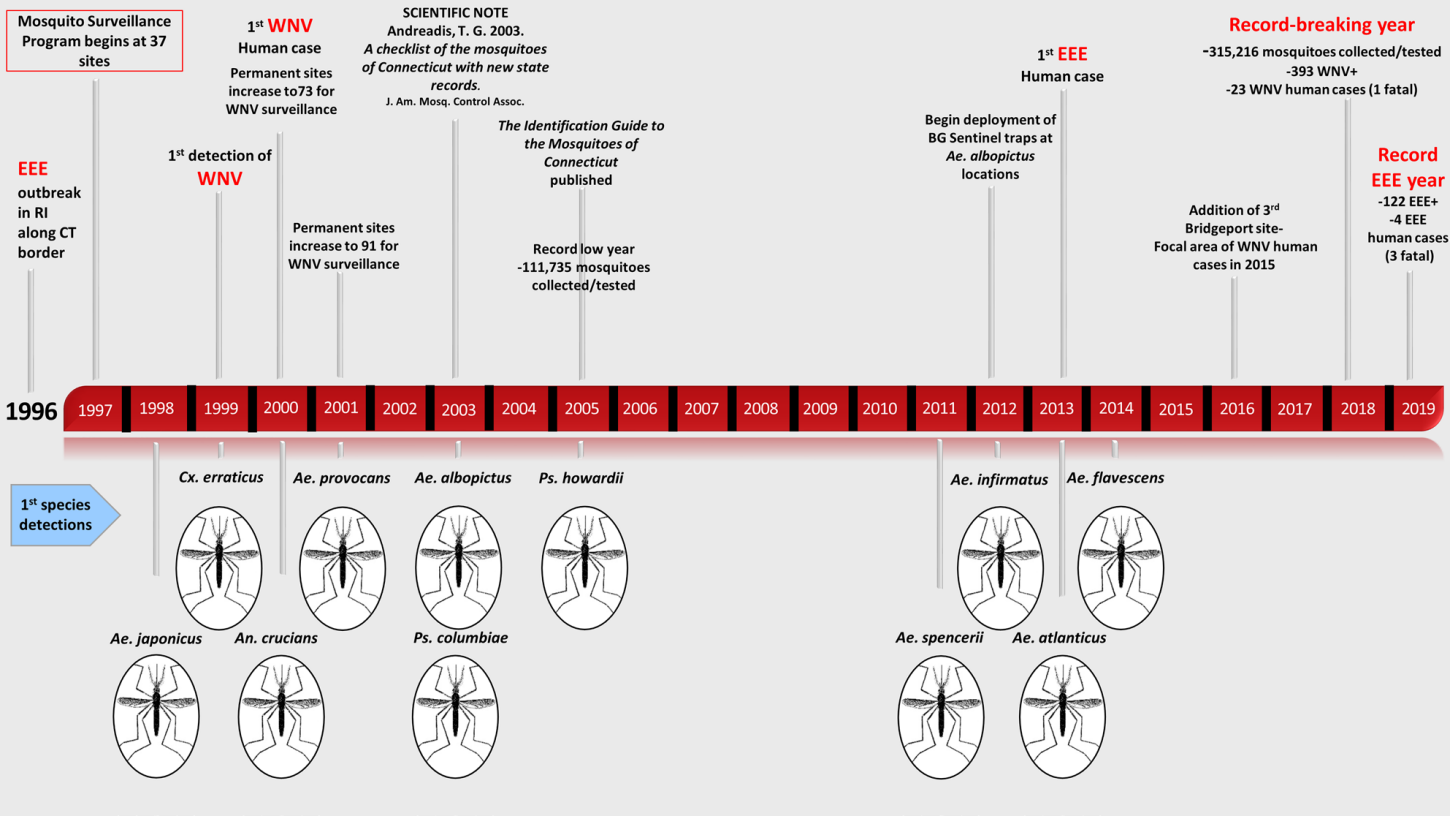
Timeline of The Connecticut Agricultural Experiment Station’s (CAES) mosquito and arbovirus surveillance network, 1996–2019. The top portion of the timeline identifies significant events in the development of the network with special mention of published reports of mosquito communities in the state. The bottom portion identifies year of first detection for 11 invasive and range expansion mosquito species detected through the surveillance network. The figure was created in Microsoft PowerPoint 2016 with images created by CAES.

Trends in species evenness were negative using both GLMMs (“Year”—Est. − 0.01, t-value − 7.86) and GAMMs (“Year (centered)”—Est. − 0.04, t-value − 5.58, p = 0.000) (Fig. 2D): this trend resulted in a predicted 12% decrease in site-level species evenness from 2001 to 2019. Similar to fixed effects GLMMs of species richness, fixed effects GLMMs of species evenness were less informative than a random effects-only model (ΔAIC 66.5). Declining intercept values were evident when evaluating “Year” as a random effect (S. Fig. 5), further supporting an overall annual decline in species evenness estimates among sites.

##### Spatial regressions

Similar to spatial models of species richness, GLMMs predicted positive relationships between species evenness and coordinate (Latitude (centered): Est. 0.36, t-value = 7.63; Longitude (centered): Est. 0.18, t-value = 8.54); the best fitting GAMM, which included Longitude by Latitude smoothing terms, also predicted positive relationships (Smoothing term 1: Est. 0.12, p = 0.01; Smoothing term 2: Est. 0.16, p = 0.004). GAMM predictions of site-level species evenness were equally complex to predictions of abundance and richness, and predicted evenness to be highest in southcentral and eastern CT (S. Fig. 6). Fixed effect GLMMs provided very poor fits to the data compared with random effects-only models (Latitude: ΔAIC 502.6; Longitude: ΔAIC 488.4; see the above results for *Site-level collections: spatial regressions* for possible reasons for these poor fits).

##### Weather correlations

We did find a negative correlation between statewide prior spring minimum temperatures and mosquito species evenness (r = − 0.49, − 0.77 to − 0.04).

##### Land cover correlations

Positive correlations of note for species evenness included: deciduous forest (r = 0.46, 0.28–0.61) and forested wetland (r = 0.22, 0.01–0.41). Negative correlations included: barren (r = − 0.37, − 0.54 to − 0.18), developed (r = − 0.45, − 0.60 to − 0.26), and open water (r = − 0.32, − 0.50 to − 0.12).

#### Correlations between abundance, richness, and evenness

The relationships between abundance, richness, and evenness varied depending on the scale examined. Across all years of data at the site-level, the correlation between abundance and richness was positive (r = 0.53, 0.36–0.67), the correlation between abundance and evenness as negative (r = − 0.35, − 0.52 to − 0.15), and there was no correlation of note between richness and evenness. Across all sites at the year-level, there were no correlations of note between abundance, richness, and evenness. Annual statewide correlations between richness and evenness (RRE) were positive for all years yet there was no noticeable annual trend in these correlations (Fig. 2E). Spatially, the average site-level RRE was 0.15 (± 0.03 SE). Furthermore, the magnitude and direction of RRE tended to increase on a south to north gradient (r = 0.31, 0.11–0.49), yet there was no apparent relationship in RRE along a west to east gradient (S. Fig. 7). We did detect a positive correlation between RRE and average maximum spring temperatures (r = 0.46, 0.01–0.76) as well as a positive correlation between RRE and percent land cover classified as coniferous forest (r = 0.23, 0.02–0.42).

#### Single detection events

Single detection events were defined as the prevalence of single species detections at a site (i.e., number of species with a single pool divided by species richness). Changes in single species detections could indirectly indicate range expansion among species (i.e., the prevalence of single detections decreases with time) and/or areas of unique mosquito diversity (i.e., the prevalence of single detections changes across space).

##### Temporal regressions

We detected no overall pattern of increasing/decreasing annual prevalence of single-species detections among sites (GLMM, “Year”—Est. − 0.13, t-value = − 1.12, p = 0.22; GAMM, “Year”—Est. 0.02, t value = − 0.31, p = 0.75) (Fig. 2F). These models were considered equivalent to a random effects-only GLMM (ΔAIC < 2), and thus, there were no obvious temporal trend of increasing/decreasing frequency of single species detections among sites (S. Fig. 8). We did find a negative correlation between both collections and single-species detections among sites (r = − 0.81, − 0.92 to − 0.56), indicating that increases in collections are associated with increased species detections within the mosquito community.

##### Spatial regressions

Unlike all previous models, GLMM and GAMM spatial regressions of single species detections by coordinate all provided poor fits to the data and indicated no obvious linear and nonlinear trends in single species detections in CT (Fig. 1B, S. Fig. 9).

### Objective 2: annual collections of mosquito populations among species

Our second objective was to identify species-level linear and nonlinear annual collection trends that would suggest growth or decline in mosquito community composition. Because data were aggregated across the state to the species-level in these analyses, we did not perform any spatial regressions with this data. All regression results and tables are provided as supporting information in *Supporting Information: Regression Tables*.

#### Total abundance

##### Temporal regressions

Annual trends in total abundance per mosquito species were positive (GLMM “Year” Est. 0.05, t-value 7.59; GAMM “Year” Est. 0.33, t-value 2.85, p = 0.0045), with a predicted doubling of per-species annual collections in CT from 2001 to 2019 (Fig. 3A). Fixed effects models of species-level collections were less informative than random effects-only models (ΔAIC 11.2); however, there was a clear pattern of increasing “Year” random intercept estimates in the null model (S. Fig. 10), further supporting an increase in abundance across species.

#### Single detection events

Among species, our regressions identified a linear (GLMM, “Year”—Est. − 0.03, t-value = − 8.47) and non-linear (GAMM, “Year”—Est. − 0.02, t-value = − 3.35, p = 0.0009) decline in single-site detections (Fig. 3B). In these models of single-site detections among species, fixed effects models provided a poor overall fit to the data compared to random effects-only models (ΔAIC 145.6). There was also a strong temporal pattern of decline in the intercept estimates for “Year” when “Year” was evaluated as a random effect (S. Fig. 11), further suggesting a pattern of spatial growth (i.e., a decline in single-site detections) among mosquito species in CT.

We did find a negative correlation between species-level collections and the proportion of single site detections among species (r = − 0.81, − 0.92 to − 0.56), indicating that increases in collections are associated with increased spatial detections (i.e., declines in single species detections).

#### Species-specific trends

Since 2005, five additional species have been documented in CT (Table 1), including *Aedes atlanticus* (Dyar and Knab)*, Aedes flavescens* (Muller)*, Aedes infirmatis* (Dyar and Knab)*, Aedes spencerii* (Theobold)*,* and *Psorophora howardii* (Coquillett). Initial detections of each species were along the southern border of CT (Fig. 4). We further identified nine species possibly undergoing population increases in CT: *Ae. albopictus*, *Aedes taeniorhynchus* (Wiedemann)*, Anopheles crucians* (Wiedemann), *Anopheles quadrimaculatus* (Say)*, Anopheles walkeri* (Theobald)*, Cx. erraticus*, *Culex territans* (Walker)*, Psorophora columbiae* (Dyar and Knab), and *Ps. howardii* (Table 1, Fig. 4). Many of these novel and expanding species tended to display a more southern distribution (Fig. 4), suggesting that many of the species possibly experiencing population expansions are moving from south to north. We found further evidence of a south to north expansion of species populations when examining correlations between total site-level species richness and latitude (r = − 0.29, − 0.47 to − 0.08).

**Table 1:** New, emerging, and declining mosquito species in Connecticut, U.S. 2001–2019.

Designation	Species	1st State Record	Peak year	Total	Total towns	Evidence of growth/decline	Virus detection^+^	Host preference^++^
New species since 2005	*Aedes atlanticus*	2014	2018	42	15	–	–	Mammalian
	*Aedes flavescens*	2014	–	1	1	–	–	Mammalian
	*Aedes infirmatus*	2012	2018	30	9	–	–	Mammalian
	*Aedes spencerii*	2011	–	1	1	–	–	Mammalian
	*Psorophora howardii*	2005	2018	142	26	Growth	–	Mammalian
Expanding Species (defined as increases in collections and detections)	*Aedes albopictus*	2003	2018	10,398	31	Growth	CVV*, PTV*, WNV*	Mammalian
	*Aedes taeniorhnychus*	Native to region	2011	150,134	38	Growth	CVV*, JCV*, PTV*, WNV*	Mammalian
	*Anopheles crucians*	Native to region	2019	2,234	51	Growth	CVV*, JCV*, EEEV*, WNV*	Mammalian
	*Anopheles quadrimaculatus*	Native to region	2015	1,122	63	Growth	CVV*, EEEV*, HJV*, JCV*, PTV*, WNV*	Mammalian
	*Anopheles walkeri*	Native to region	2014	7,180	52	Growth	CVV*, EEEV*, JCV*, PTV*, WNV*	Mammalian
	*Culex erraticus*	1999	2019	1,178	39	Growth	–	Avian, reptilian
	*Culex territans*	Native to region	2017	2,790	71	Growth	WNV*	Avian, reptilian
	*Psorophora columbiae*	2003	2018	96	18	Growth	–	Mammalian
Declining Species (defined as declines in collections and detections)	*Aedes trivittatus*	Native to region	2011	204,120	74	Decline	CVV*, EEEV*, HJV*, JCV*, PTV*, TVT*, WNV*	Mammalian
	*Aedes sticticus*	Native to region	2003	18,590	73	Decline	EEEV*, JCV*, PTV*, TVT*, WNV*	Mammalian

New species include individuals collected through any mosquito surveillance method (standard or exploratory) and site (standard and supplemental) employed by the Connecticut Agricultural Experiment Station’s mosquito and arbovirus surveillance network. Expanding and Declining species are determined using linear regressions of collections and detections using only data from CO_2_-baited CDC light traps at 87 standardized sites in CT sampled from 2001 to 2019.

*Denotes confirmed isolates from CT mosquito surveillance network: *CVV *Cache Valley virus, *EEEV *eastern equine encephalitis virus, *HJV *Highlands J virus, *JCV *Jamestown Canyon virus, *PTV *Potosi virus, *TVT *Trivittatus virus, *WNV *West Nile virus.

^+^For virus isolation studies, please see^29,54–56^.

^++^For mosquito host preference studies, please see^7,26,57^.

We also detected two possibly declining species, *Aedes trivittatus* (Coquillett) and *Aedes sticticus* (Meigen) (Table 1, Fig. 4). Outside of the species listed in Table 1, eight species displayed statistical evidence of growth in either total annual collection or total spatial detections while eight species displayed statistical evidence of declines in collections or detections (see *Supporting Information: Regression Tables*). Due to the lack of evidence of growth/decline in both collections and detections for these 16 species, we did not make any conclusions as to whether these species were actually increasing or decreasing in the state.

## Discussion

We used multiple statistical approaches to quantify changes in mosquito community composition in Connecticut, U.S. since 2001. Overall, in 19 years of surveillance total mosquito collections have grown annually and five species, including *Ae. atlanticus, Ae. flavescens, Ae. infirmatus, Ae. spencerii* and *Ps. howardii*, were identified for the first time since 2005, representing a 10.2% increase in mosquito species richness. In addition to these five novel species, collections of eight other species likely grew throughout CT, including *Ae. albopictus, Ae. taeniorhynchus, An. crucians, An. quadrimaculatus, An. walkeri, Cx. erraticus, Cx. territans*, and *Ps. columbiae.* All novel/expanding species are capable of generating multiple generations in a year, eleven utilize mammalian species as blood meal sources, and seven displayed a pattern of northward expansion. We identified a spatial trend of mosquito species richness with total mosquito species richness being highest in the southern portion of CT while mosquito species evenness was highest in the northern/eastern portion of CT (Table 2). In summary, several mosquito species are experiencing range shifts, northward expansions, and population growth in the northeast region of the U.S. which may have potentially important impacts on arbovirus transmission in the region^29^.

**Table 2:** Summary results from site-level analyses of annual collections, mosquito species richness, and mosquito species evenness by time, climate, land cover, and spatial location.

Site-level variable	Year (GLMMs)	Climate variables (correlation)	Land cover (correlation)	Spatial orientation (GAMMs)
Annual abundance	Increasing	*Temperature* None	*Positive*: barren, forested wetland, non-forested wetland	Latitude: positive, increasing from south to north
		*Precipitation* Summer: positive	*Negative*: grass	Longitude: positive, increasing from west to east
Annual species richness	Increasing	*Temperature* None	*Positive*: forested sites (coniferous, deciduous, wetland)	Latitude: positive, increasing from south to north
		*Precipitation* None	*Negative*: barren, developed, grass, open water	Longitude: positive, increasing from west to east
Annual species evenness	Decreasing	*Temperature* Spring minimum: negative	*Positive*: deciduous and forested wetlands	Latitude: positive, increasing from south to north
		*Precipitation* None	*Negative*: barren, developed, open water	Longitude: positive, increasing from west to east
Annual richness/evenness correlation	No trend	*Temperature* Spring maximum: positive	*Positive:* coniferous forest	Latitude (correlation): positive, increasing from south to north
		*Precipitation* None	*Negative:* None	Longitude (correlation): none

We found multiple forms of statistical evidence that indicate an expansion of mosquito species populations in CT in the previous two decades. This expansion has occurred in conjunction with a trend of warming temperatures; however, we found no correlations between annual collections/species richness and summer/spring/winter temperatures. This expansion has also occurred in conjunction with a general annual drying trend, despite detecting a positive correlation between summer precipitation and total mosquito collections. This correlation likely reflects the general dependence of multiple mosquito species on aquatic habitats, and the capacity of populations to grow rapidly in response to extreme precipitation events^31–33^. Importantly, our analyses of climatic variables and mosquito community composition focused on state-level comparisons predominately due to a lack of available site-specific climatological data. Mosquitoes are acutely sensitive to temperature, humidity, and precipitation at very fine spatial scales, and as such, our analyses ignored such fine-scale variation in climate. Additionally, throughout our analyses our random effects-only GLMMs outperformed fixed effect GLMMs, clearly suggesting that both site- and year-specific factors explain a large amount of variance in our mosquito community composition analyses. Other factors unexamined in this report, such as variability of extreme weather events, site-specific microclimate, and the interactions between these and other factors, could be influencing patterns of mosquito community collections across the state.

Our correlations of mosquito collections and land cover support previous short-term research on mosquito communities^13–18^: after accounting for surveillance effort, richness and evenness were highest in sites with a greater prevalence of forested land cover and lowest in sites with a greater prevalence of land cover associated with human developments. This trend in richness was further evident in our GLMMs of collections by latitudinal coordinate (which also reflect the dominant land cover gradient of urban to forested in CT). Across all trapping data, total richness was highest along the southern boundary of CT while evenness tended to increase with latitude as well as longitude. The overall greater species richness in southern CT supports the view that many mosquito species are expanding northward, possibly in response to climate change^20,28,34^. The spatial pattern of total species evenness also likely reflects patterns of land cover which becomes increasingly forested moving from southwest to northeast in CT. Despite these coarse-level correlations, it is important to note that land cover changes likely play only a marginal role in mosquito community growth as land cover has changed little in CT since 1985 (https://clear.uconn.edu/index.htm; between 1985 and 2015, there has been an average of 3.5% increase in developed, 1.5% increase in turf/grass, 3.8% decrease in forested, and − 1.4% decrease in agricultural land cover types across CT). Further research pertaining to local dynamics of mosquitoes, weather patterns, and inter and intraspecific interactions are needed to better distinguish the influence of climate, predation, and competition on the expansion of mosquito populations in CT.

One unique aspect of our analyses was our investigation of the relationship between mosquito species richness and evenness (i.e., RRE). There is debate in the ecological literature as to whether variability in richness and evenness is driven by (in)dependent mechanisms^35^, and distinguishing the relationship between richness and evenness can have important implications for biodiversity research and conservation. Spatial and temporal patterns of richness and evenness were opposite, which suggests there should be no significant relationship, i.e. RRE ~ 0, between the two metrics^35^; however, we did detect a latitudinal trend of increasing RRE (both in terms of magnitude and direction). This could be an effect of the dominant gradient between urban-forested habitats in CT and the associated patterns detected between land cover and community composition metrics, with forested environments more prevalent in the eastern and northern portion of CT. Importantly, each metric measures a different aspect of community assembly, yet in the context of mosquito-borne pathogens, species richness is most often examined in relation to patterns of pathogen prevalence/incidence^17,18,29,36^. Further research is needed to determine the utility of measuring species evenness in relation to pathogen transmission.

In terms of our species-level analyses, we found inconsistent patterns of growth across all species in CT. The lack of a common trend among all species was not unexpected, as species display different life history traits and share unique relationships with habitat and climate, and thus are unlikely to respond uniformly to changes in either factor. We did, however, detect multiple indicators that mosquito species populations are mostly expanding in CT. First, we detected a decrease in single-site detections across species. We also documented expanding detections of multiple novel, range expansion, and invasive species along a south to north latitudinal gradient while not detecting any north to south movement of species. Our analyses of species-specific patterns of growth further identified commonalities among species experiencing expansions in CT, including multiple generations per year and a larval habitat preference for temporary (temporary flooded ground pools or containers) and (semi)permanent high-water habitats (swamps and bogs); most expanding species also display mammalian host blood feeding preferences^37,38^. Opportunistic traits have been previously identified as a signature of successful invasive or range expansion mosquito species^39^, and our surveillance results extend this result beyond the invasive container breeders such as *Ae. japonicus* and *Ae. albopictus* to include such species as *Ae. atlanticus, Ps. columbiae,* and *Ps. howardii*. The invasion dynamics of *Ae. japonicus* and *Ae. albopictus* and their ability to vector arboviruses have been widely discussed, and these species are currently established in CT^28,40,41^.

The growth in collections of common species such as *Ae. taeniorhynchus, An. crucians, An. quadrimaculatus, An. walkeri, Cx. erraticus,* and *Cx. territans* that are common in (semi)permanent water-body habitats may be consistent with a trend of restoration of historical wetland habitats in the northeast region of the U.S.^42^. *Culex erraticus* has been implicated as an enzootic vector of EEEV^43,44^, though it remains to be seen if *Cx. erraticus* will (or has) contributed to transmission of any arbovirus in CT^29^; *Anopheles quadrimaculatus* was also the historical primary vector of malaria transmission in the U.S. Growth in *Ae. taeniorhynchus* collections and the establishment of *Ps. columbiae*, two nuisance species known to aggressively bite humans, could impact the public’s enjoyment of certain environments such as human developments near salt-water marshes (*Ae. taeniorhynchus*) and golf courses (*Ps. columbiae*). While our regressions of these species’ annual collections indicate possible population growth, further research is needed on each species to better define the likelihood of these results. We also further caution over-interpreting possible expansions of arboviral transmission with the expansion of the species identified in Table 1 as none of these listed species are the likely primary vectors of any arbovirus currently under surveillance in CT^29^.

We did document a possible decline for *Aedes sticticus and Aedes trivittatus. Aedes sticticus* is a river floodplain species that relies on flooding alongside rivers and streams to provide larval habitat. Possible habitat factors affecting the population of *Ae. sticticus* include channelization of streams due to urbanization and the legacy of waterway damming in the northeast; whether the statewide effort to rehabilitate river floodplains and remove unnecessary and defunct dams in CT could result in a rebound of *Ae. sticticus* is uncertain^45^. *Aedes trivittatus* is often associated with temporarily flooded ground level depressions and is considered a localized nuisance species in a number of habitats throughout the northeast. This species’ decline could be linked to the overall decline in summer precipitation observed in CT; however, other aggressive human-biting species that display a similar life history strategy, such as *Aedes vexans* (Meigen) and *Psorophora ferox* (Humbolt), showed no trends of note. While no one likely laments the decline of a mosquito species, future studies on *Ae. sticticus* and *Ae. trivittatus* could elucidate ecological mechanisms of decline that could be incorporated into integrated mosquito management practices.

## Conclusion

Mosquitoes are an important grouping of insect species that, due to their ability to vector pathogens among humans and between humans and wildlife, require constant surveillance. The resulting datasets produced from these efforts provide long-term data to test the generalizability of insect declines that have been observed in other taxa. We have shown that in the northeast region of the U.S., overall mosquito abundance has increased annually and there have been multiple introductions of native and invasive species into the region in the previous 20 years. We also identified commonalities among species experiencing growth and expansion in the region: opportunistic egg-laying behaviors, a reliance on (semi)permanent bodies of water, and a preference for mammals as blood meal hosts. Overall, we detected a south-to-north trend of increasing community richness, indicating that many species are moving northward, possibly in response to changes in land use and climate. How these changes in mosquito community composition will impact mosquito-borne pathogen transmission in the future will require further eco-epidemiological investigations.

## Methods

### Mosquito surveillance in Connecticut

The Connecticut Mosquito Trapping and Arbovirus Surveillance Program has been operational since 1997. Initially, 37 trapping locations were selected for eastern equine encephalitis virus (EEEV) surveillance. In 2000 and 2001, the program was expanded to 73 and 91 locations respectively, due to increased mosquito trapping and arbovirus surveillance for WNV. Ninety-one sites were standardized in 2004 with an additional trapping site added in 2016 (Fig. 5); supplemental trapping locations have been added on a seasonal basis to evaluate elevated risk of transmission of WNV or EEEV to the public as appropriate. Beginning in 2012, Biogents BG Sentinel traps, baited with the Human Scent Lure, have been utilized for increased surveillance of *Ae. albopictus*, primarily at locations where this invasive species has been detected from 2012 to 2019. Each surveillance season traditionally begins the first week of June and continues through the end of October. Throughout each season, mosquitoes are collected at each site using a single CDC light trap baited with CO_2_ (as dry ice) and a single gravid trap baited with a hay-lactalbumin infusion. Traps are set late morning or early afternoon and collected early in the morning the following day. Surveillance is conducted at each site approximately once every 10 days; if there are isolations of WNV or EEEV, trapping is then conducted once or twice a week at those sites for the remainder of the surveillance season. All collections are tested for arboviral infections using cell culture and RT-PCR with a suite of arbovirus primers^46^.

### Trend analyses

We limited our trend analyses to trap sites that have remained in continuous operation between 2001 and 2019 (n = 87 sites). We then further limited our analyses to collections from ground-level CO_2_-baited light traps only; this is because light traps are the least biased collection device used by the network^6^. Because the intent of this manuscript was to investigate changes in mosquito community composition, we did not examine trends in arboviral detections as have been previously reported^29^.

### Objective 1: annual collections of mosquito populations among sites

The first objective of our study was to identify temporal and spatial trends in annual collections of mosquito populations among sites. We examined linear and nonlinear (i.e., smoothing) trends in total annual collections, species richness, species evenness, and the prevalence of single-species detections using generalized linear/additive mixed effects models (GLMMs and GAMMs, respectively).

For spatial and temporal models of total collections, the response term was the log-transformed total annual collection per site, trapping effort (defined as the number of trap nights at each individual site per year) was an intercept-offset term (GLMMs) or a fixed effect term (GAMMs), year/latitude/longitude were individual fixed linear (GLMMs) or smoothing (GAMMs) terms, and either trap site (temporal regressions) or year (spatial regressions) were random intercept effect term; we also explored interactions between spatial coordinates in the GAMMs. A natural log transformation was used to investigate trends in collections among sites due to the large variance in total collections observed among these terms and to improve model convergence. In the spatial regressions, latitude and longitude were centered to their average value in the data set in order to improve model convergence. We chose to model sites and years as random effects to account for inherent similarities of repeated measures as well as to better evaluate variation among these variables^47^.

For models of species richness and evenness, richness and evenness were first calculated using the *specnumber* and *diversity* functions available in the R package “vegan”^48^. In all GLMMs/GAMMs of richness/evenness, richness/evenness and year/coordinate were centered to the average values in order to improve model convergence. For models of the annual proportion of single species detections among sites, changes to the above methodology included modeling the response term as a binomial-error distributed proportion and centering Year or coordinate. These models essentially examined patterns in the detection of “singletons”, a term which describes “rare” species detections and has been used in the conservation literature to identify areas with an overabundance of rare species (which may in turn identify areas with unique ecologies for conservation). In this report, our aim was to identify whether single detections events increased or decreased with time (which may provide additional evidence of community changes) rather than to identify regions of unique mosquito species diversity. Because CAES records and tests collected mosquitoes for arboviruses as pooled samples, we defined singletons as the sole pool of a particular species identified at a site in a single year.

We examined coarse-scale correlations in state-wide annual collections and seasonal weather variables as well as site-wide total collections and land cover classifications using the Pearson’s correlation coefficient; all state-wide and site-wide metrics were corrected for trapping effort in these analyses. Weather comparisons included within season comparisons of minimum, average, and maximum seasonal temperature and total precipitation (data aggregated from May to October) as well as comparisons between prior Winter (December of prior year–February) and prior Spring (March–May) and total seasonal collections. Daily weather data in Connecticut from 2001 to 2019 were obtained from the National Oceanic and Atmospheric Administration’s Climate Date Online platform (n = 42 stations). Land cover data was obtained from UCONN CLEAR (https://www.clear.uconn.edu) as reported previously^29^. Briefly, a 0.51 km buffer was drawn around the geolocation of each trap site in ArcMap 10.5.1, land cover attributes were clipped to this buffer, and the percentage of each land cover classification was calculated. Because land cover has changed very little in CT since 1985 (https://www.clear.uconn.edu), we performed correlations between percentage land cover derived from the 2015 land cover data and the total collection at each trap across all years corrected for total trapping effort across all years . Finally, we examined the correlation between richness and evenness among sites and across the state using the Pearson correlation coefficient (termed RRE in community ecology analyses^35^).

We used the *glmer* and *lmer* function in the “lme4” R package for all GLMMs^49^ and the *gamm* function in the “nlme” R package for all GAMMs^50^. To facilitate model comparisons between GLMM random effect models and fixed effect models, maximum likelihood estimation was employed in all GLMMs (i.e., coding REML = FALSE). Model predictions, random effects, and residuals were assessed using the *ggpredict*, *plot_model*, and *qqnorm()* functions available in the “ggeffects” R package, “sjPlot” R package, and base R, respectively^51,52^. All models described above were compared to random effect-only models using the Akaike Information Criteria (AIC). Correlations were performed using *cor.test()*, which is available in base R^53^.

### Objective 2: annual collections of mosquito populations among species

We repeated only the temporal regressions listed above for our analyses of species-level collections. In regressions of collections by year among species, the response term was the log (+ 1)-transformed total annual collection per species, annual trapping effort was an intercept-offset term (GLMMs) or a fixed term (GAMMs), year was a fixed linear (GLMMs) or smoothing (GAMMs) term, and species was a random intercept effect term. A natural log (+ 1) transformation was used to investigate trends in collections among species due to the large variance in total collections observed among these terms and to improve model convergence; the (+ 1) was included because some species were not collected in all years. Similar to models described above for single species detections among sites, we examined the annual proportion of single site detections among species using GLMMs and GAMMs. However, both the response term (single-site detection) and predictor term (year) were centered to improve model convergence.

We completed our among species analyses by identifying species-specific trends that may signify possible population growth or decline in a species since the 2005 publication of the identification guide of mosquitoes in the state of CT^26^. First, we utilized all data available from all trap types within the CAES dataset to identify all novel species detections. We then analyzed either total annual collections (log transformed) or number of sites detected for each species with a simple linear regression with year as the predictor variable and annual trapping effort as an intercept offset; a Poisson-error generalized linear regression was implemented for regressions of number of sites detected. Evidence for growth in collections and spatial detections were used to identify which species’ populations likely expanded in the state in the previous 20 years; likewise, evidence for declines in collections and spatial detections were used to identify which species’ populations are likely declining in the state.

## Supplementary information

Supplementary Figures.

Supplementary Tables.

Supplementary Data.

## Data Availability

All data necessary to reproduce these results are included as Supporting Information.
