# Supplementary material for: Increased mosquito abundance and species richness in Connecticut, United States 2001–2019
Source: Sci Rep. 2020 Nov 6;10:19287. doi: 10.1038/s41598-020-76231-x (PMC7648108; doi:10.1038/s41598-020-76231-x)
Supplement: Supplementary file 1 — Supplementary Figures. [file 41598_2020_76231_MOESM1_ESM.docx]

**Supporting Figures for:**

Increased Mosquito Abundance and Species Richness in Connecticut, United States 2001 - 2019

**By the following authors:**

Tanya Petruff*, Joseph R McMillan*, John Shepard, Theodore G Andreadis, Philip M Armstrong,

*Co-first authors

**Author Affiliations:**

Center for Vector Biology and Zoonotic Diseases, Environmental Sciences, Connecticut Agricultural Experiment Station, New Haven, Connecticut

**Corresponding Author contact:**

Joseph R McMillan

The Connecticut Agricultural Experiment Station

123 Huntington Street

New Haven, CT 06511

203-974-8515 (w); 678-642-4618 (c)

[Joseph.McMillan@ct.gov](mailto:Joseph.McMillan@ct.gov)


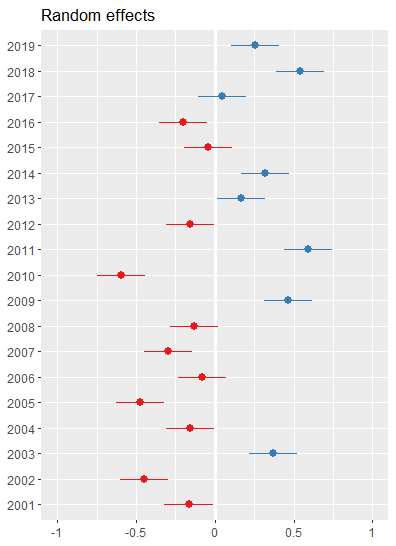


**S. Figure 1.** Year-level random effect estimates from a linear mixed effects model with site-level annual mosquito collections (natural log transformed) as the response variable, trapping effort (natural log transformed) as an intercept offset term, and year and site as random intercept terms. Blue indicates a positive increase in the model’s intercept while red indicates a negative change in the model’s intercept. Points represent the estimate while lines represent the 95% confidence interval of the estimate.


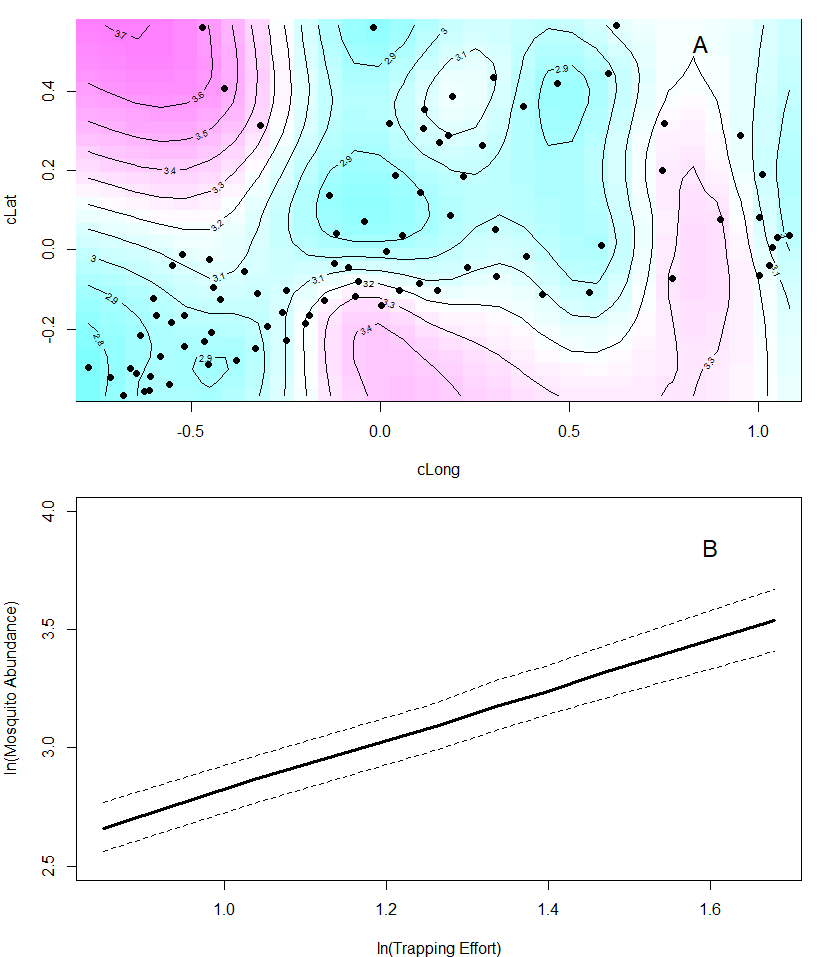


**S. Figure 2**. A) The predicted site-level mosquito abundance (natural log transformed) by longitude and latitude coordinate (each centered to the corresponding average value). Points represent the surveillance site location, lines represent gradients of predicted abundance with colors further emphasizing the predictions (blue – low, pink – high). B) The predicted (solid line, 95% CI dashed line) relationship between site-level trapping effort and site-level mosquito abundance. Predictions were generated from a general additive mixed effects model with site-level annual collections (natural log transformed) as the response variable, trapping effort (natural log transformed) as a fixed effect term, longitude by latitude (each centered) as a smoothing term, and year of collection as a random effect.

**S. Figure 3**. Year-level random effect estimates from a linear mixed effects model with site-level annual mosquito species richness (Centered to average value), trapping effort (natural log transformed) as an intercept offset term, and year and site as random intercept terms. Blue indicates a positive increase in the model’s intercept while red indicates a negative change in the model’s intercept. Points represent the estimate while lines represent the 95% confidence interval of the estimate.


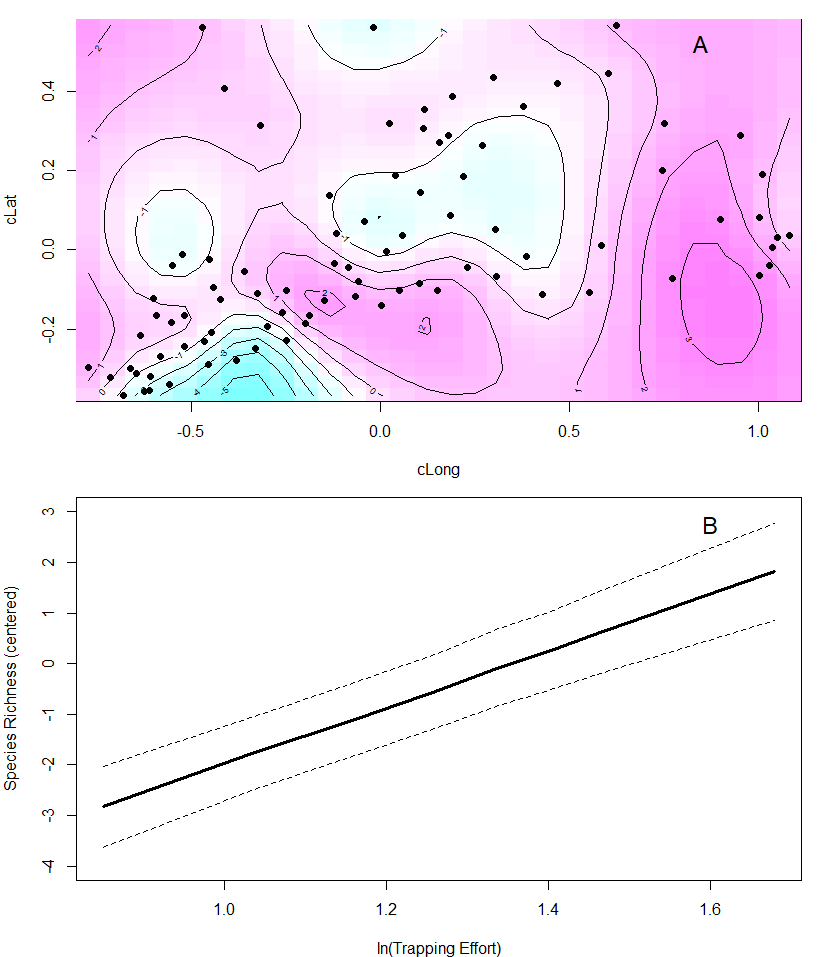


**S. Figure 4.** A) The predicted site-level mosquito species richness (centered) by longitude and latitude coordinate (each centered to the corresponding average value). Points represent the surveillance site location, lines represent gradients of predicted abundance with colors further emphasizing the predictions (blue – low, pink – high). B) The predicted (solid line, 95% CI dashed line) relationship between site-level trapping effort and site-level mosquito species richness. Predictions were generated from a general additive mixed effects model with site-level annual species richness (centered) as the response variable, trapping effort (natural log transformed) as a fixed effect term, longitude by latitude (each centered) as a smoothing term, and year of collection as a random effect.


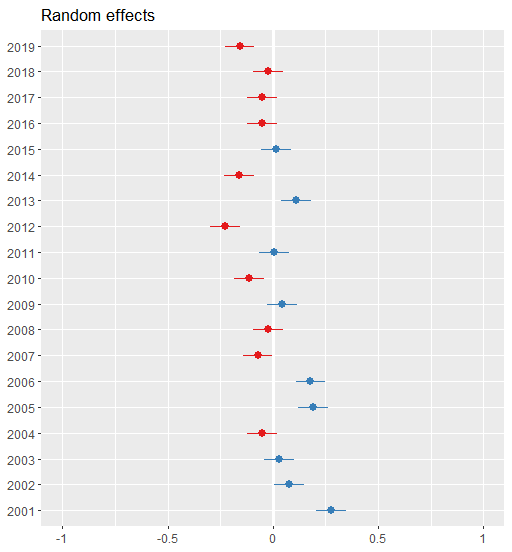


**S. Figure 5**. Year-level random effect estimates from a linear mixed effects model with site-level annual mosquito species evenness (Centered to average value), trapping effort (natural log transformed) as an intercept offset term, and year and site as random intercept terms. Blue indicates a positive increase in the model’s intercept while red indicates a negative change in the model’s intercept. Points represent the estimate while lines represent the 95% confidence interval of the estimate.


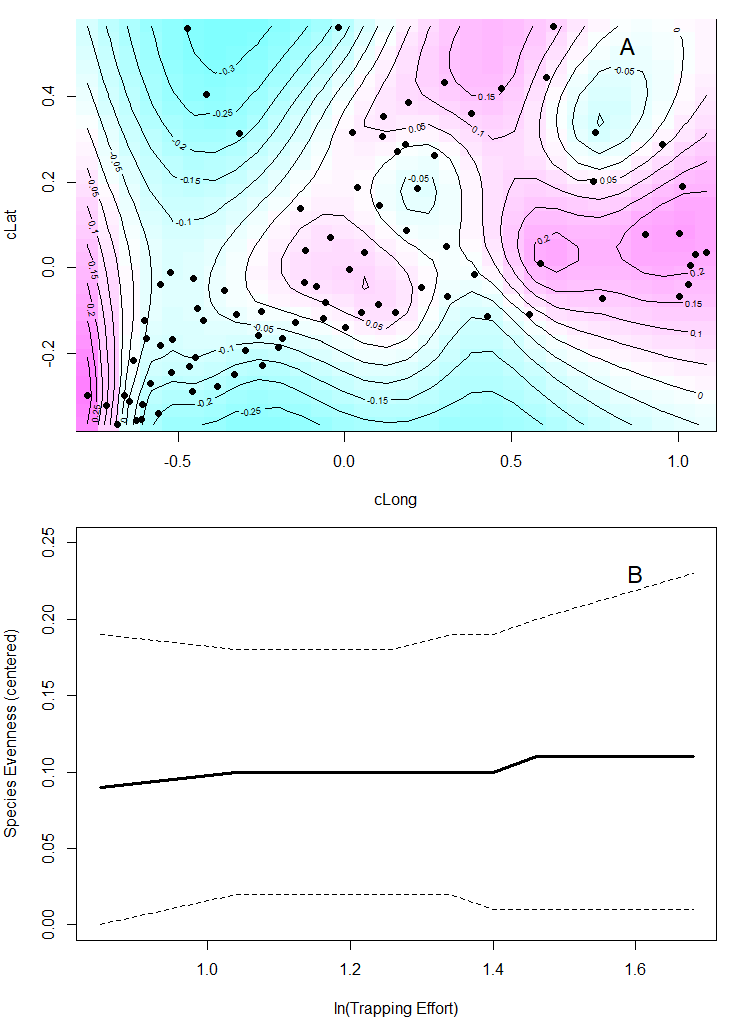


**S. Figure 6.** A) The predicted site-level mosquito species evenness (centered) by longitude and latitude coordinate (each centered to the corresponding average value). Points represent the surveillance site location, lines represent gradients of predicted abundance with colors further emphasizing the predictions (blue – low, pink – high). B) The predicted (solid line, 95% CI dashed line) relationship between site-level trapping effort and site-level mosquito species evenness. Predictions were generated from a general additive mixed effects model with site-level annual species evenness (centered) as the response variable, trapping effort (natural log transformed) as a fixed effect term, longitude by latitude (each centered) as a smoothing term, and year of collection as a random effect.


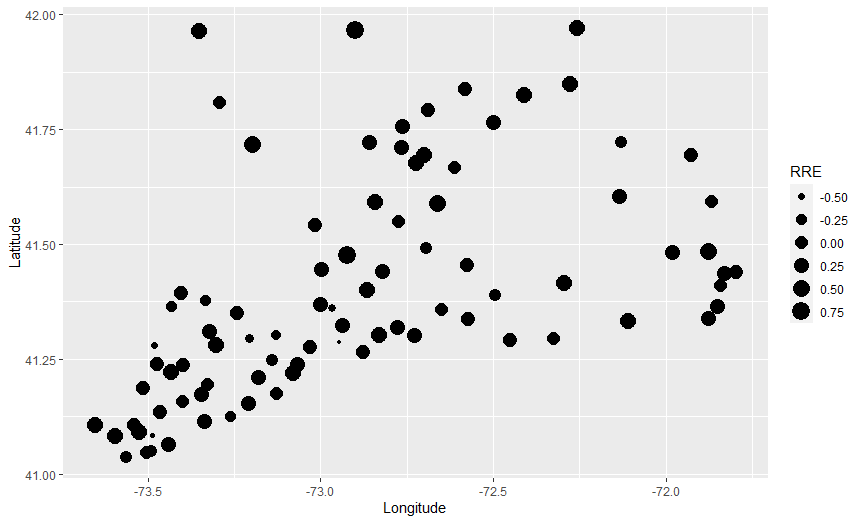


**S. Figure 7.** The spatial distribution of the site-level correlation between mosquito species richness and mosquito species evenness in Connecticut, U.S. Data comes from mosquito collections in CO_2_-baited light traps set at 87 locations from 2001 – 2019.

**S. Figure 8.** Year-level random effect estimates from a binomial error mixed effects model with the proportion of single species detections among sites as the response variable, trapping effort (natural log transformed) as an intercept offset term, and year and site as random intercept terms. Blue indicates a positive increase in the model’s intercept while red indicates a negative change in the model’s intercept. Points represent the estimate while lines represent the 95% confidence interval of the estimate.


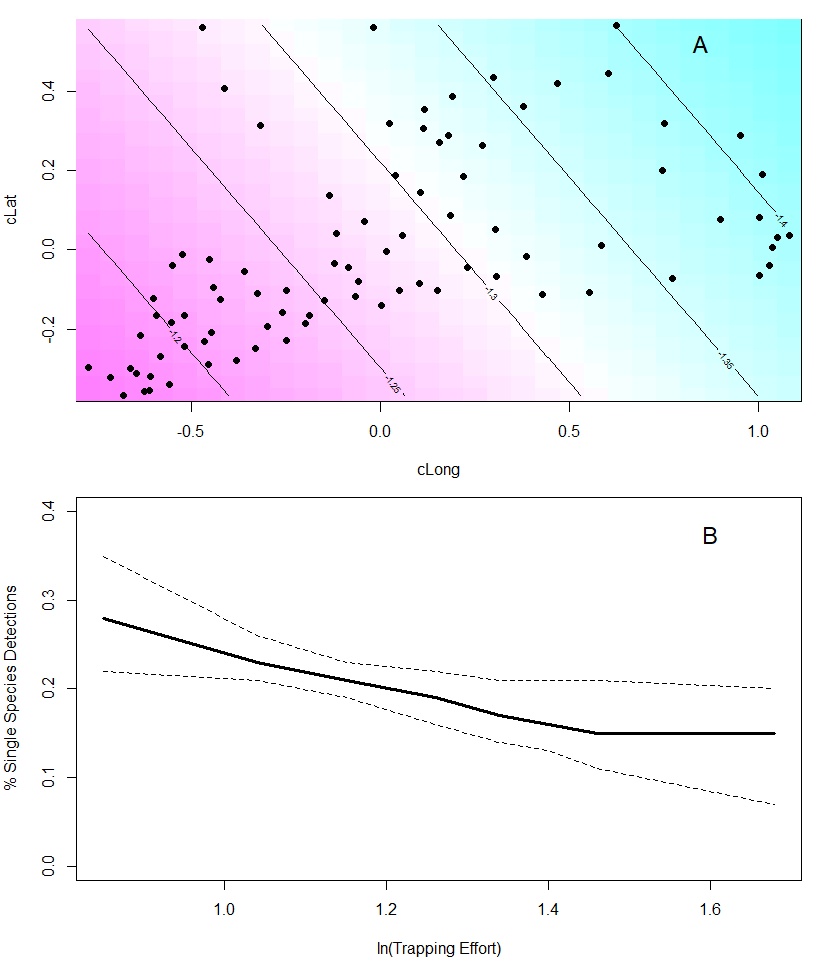


**S. Figure 9**. A) The predicted site-level prevalence of single species detections by longitude and latitude coordinate (each centered to the corresponding average value). Points represent the surveillance site location, lines represent gradients of predicted abundance with colors further emphasizing the predictions (blue – low, pink – high). B) The predicted (solid line, 95% CI dashed line) relationship between site-level trapping effort and site-level single species detections. Predictions were generated from a general additive mixed effects model with site-level annual prevalence of single species detections as the response variable, trapping effort (natural log transformed) as a fixed effect term, longitude by latitude (each centered) as a smoothing term, and year of collection as a random effect.

**S. Figure 10**. Year-level random effect estimates from a linear mixed effects model with species-level annual collections (natural log transformed) as the response variable, trapping effort (natural log transformed) as an intercept offset term, and year and site as random intercept terms. Blue indicates a positive increase in the model’s intercept while red indicates a negative change in the model’s intercept. Points represent the estimate while lines represent the 95% confidence interval of the estimate.

**S Figure 11.** Year-level random effect estimates from a binomial error mixed effects model with the proportion of single site detections among mosquito species as the response variable, trapping effort (natural log transformed) as an intercept offset term, and year and site as random intercept terms. Blue indicates a positive increase in the model’s intercept while red indicates a negative change in the model’s intercept. Points represent the estimate while lines represent the 95% confidence interval of the estimate.
