# Supplementary material for: Increased mosquito abundance and species richness in Connecticut, United States 2001–2019
Source: Sci Rep. 2020 Nov 6;10:19287. doi: 10.1038/s41598-020-76231-x (PMC7648108; doi:10.1038/s41598-020-76231-x)
Supplement: Supplementary file 2 — Supplementary Tables. [file 41598_2020_76231_MOESM2_ESM.docx]

**Supporting Tables and Regression Results for:**

Increased Mosquito Abundance and Species Richness in Connecticut, United States 2001 - 2019

**By the following authors:**

Tanya Petruff*, Joseph R McMillan*, John Shepard, Theodore G Andreadis, Philip M Armstrong,

*Co-first authors

**Author Affiliations:**

Center for Vector Biology and Zoonotic Diseases, Environmental Sciences, Connecticut Agricultural Experiment Station, New Haven, Connecticut

**Corresponding Author contact:**

Joseph R McMillan

The Connecticut Agricultural Experiment Station

123 Huntington Street

New Haven, CT 06511

203-974-8515 (w); 678-642-4618 (c)

[Joseph.McMillan@ct.gov](mailto:Joseph.McMillan@ct.gov)

**Supporting Information: Regression tables**

| **Supporting Table** | **Page** |
| --- | --- |
| CAES mosquito surveillance summary by all trap types | 3 |
| GLMM and GAMM regressions of total site-level collections by year | 4 |
| GLMM regressions of total site-level collections by site coordinate | 5 |
| GAMM regressions of total site-level collections by site coordinate | 6-7 |
| GLMM and GAMM regressions of annual site-level mosquito species richness by year | 8 |
| GLMM regressions of total site-level mosquito species richness by coordinate | 9 |
| GAMM regressions of total site-level mosquito species richness by coordinate | 10-11 |
| GLMM and GAMM regressions of annual site-level mosquito species evenness by year | 12 |
| GLMM regressions of total site-level mosquito species evenness by coordinate | 13 |
| GAMM regressions of total site-level mosquito species evenness by coordinate | 14-15 |
| GLMM and GAMM regressions of the annual prevalence of single mosquito species detections among sites by year | 16 |
| GLMM regressions of the annual prevalence of single mosquito species detections among sites by coordinate | 17 |
| GAMM regressions of the annual prevalence of single mosquito species detections among sites by coordinate | 18-19 |
| GLMM and GAMM regressions of total species-level collections by year | 20 |
| GLMM and GAMM regressions of the annual prevalence of single-site detections among species by year | 21 |
| LM regressions of species-specific collections and site detections by year | 22-23 |

| Trap Type | # OF MOSQUITOES COLLECTED |
| --- | --- |
| CDC – Light (Ground) | 3,597,754 |
| CDC - Gravid | 411,742 |
| Biogents BG Sentinel | 15,856 |
| Combined Light and Gravid | 41,735 |
| CDC - Light (Canopy) | 38,575 |
| Method unidentified | 2,502 |
| Mosquito Magnet PRO | 836 |
| Mosquito Magnet X | 313 |
| Resting Boxes | 64 |
| Hand Collected | 23 |

**S. Table 1**. Total mosquito collections by trap type from the Connecticut Agricultural Experiment Station’s mosquito and arbovirus surveillance network 1996 – 2019.

GLMM and GAMM regressions of total site-level abundance by year

| Fixed effects | Estimate (95% CI) | Std. Error | t-value |
| --- | --- | --- | --- |
| Intercept | -47.7  (-58.8, -36.5) | 5.67 | -8.4 |
| Year | 0.03  (0.02, 0.03) | 0.003 | 9.17 |
|  |  |  |  |
| Random effects | Variance | Std. Dev |  |
| Site | 0.46 | 0.67 |  |
| Residual | 0.39 | 0.63 |  |

Results from a mixed effect linear regression of the log-transformed annual abundance of all mosquitoes in light traps set at 87 trapping locations in Connecticut, U.S. from 2001 – 2019.

| Fixed effects | Estimate  (95% CI) | Std. Error | DF | t-value | p-value |
| --- | --- | --- | --- | --- | --- |
| Intercept | 4.89  (4.50, 5.27) | 0.20 | 1563 | 24.7 | 0.000 |
| Effort (log transformed) | 0.84  (0.70, 0.97) | 0.07 | 1563 | 12.00 | 0.000 |
| Year (smoothed) | 0.77  (0.21, 1.33) | 0.29 | 1563 | 2.7 | 0.007 |
|  |  |  |  |  |  |
| Random effects | Intercept | Residual |  |  |  |
| Site | 0.68 | 0.61 |  |  |  |

Results from a mixed effect additive regression of the log-transformed annual abundance of all mosquitoes in light traps set at 87 trapping locations in Connecticut, U.S. from 2001 – 2019.

GLMM regressions of total site-level collections by latitude and longitude

| Fixed effects | Estimate (95% CI) | Std. Error | t-value |
| --- | --- | --- | --- |
| Intercept | 4.46  (4.29, 4.62) | 0.08 | 55.6 |
| Latitude (centered) | 0.49  (0.31, 0.66) | 0.09 | 5.48 |
|  |  |  |  |
| Random effects | Variance | Std. Dev |  |
| Year | 0.11 | 0.34 |  |
| Residual | 0.74 | 0.86 |  |

Results from a linear mixed effect regression with log-transformed abundance as the response term, trapping effort as an intercept offset, trapping location (latitude, centered) as a predictor term, and year as a random effect.

| Fixed effects | Estimate (95% CI) | Std. Error | t-value |
| --- | --- | --- | --- |
| Intercept | 4.46  (4.29, 4.62) | 0.08 | 55.6 |
| Longitude (centered) | 0.20  (0.12, 0.28) | 0.04 | 4.78 |
|  |  |  |  |
| Random effects | Variance | Std. Dev |  |
| Year | 0.11 | 0.34 |  |
| Residual | 0.75 | 0.86 |  |

Results from a linear mixed effect regression with log-transformed abundance as the response term, trapping effort as an intercept offset, trapping location (longitude, centered) as a predictor term, and year as a random effect.

GAMM regressions of total site-level collections by latitude and longitude

| Fixed effects | Estimate  (95% CI) | Std. Error | DF | t-value | p-value |
| --- | --- | --- | --- | --- | --- |
| Intercept | 3.78  (3.34, 4.23) | 0.23 | 1631 | 16.7 | 0.0000 |
| Effort (log transformed) | 1.26  (1.10, 1.41) | 0.08 | 1631 | 15.5 | 0.0000 |
| Latitude (centered, smoothed) | -0.41  (-1.00, 0.18) | 0.30 | 1631 | -1.35 | 0.18 |
|  |  |  |  |  |  |
| Random effects | Intercept | Residual |  |  |  |
| Year | 0.33 | 0.84 |  |  |  |

Results from an additive mixed effect regression with log-transformed abundance as the response term, trapping effort as an intercept offset, trapping location (latitude, centered) as a smoothing term, and year as a random effect

| Fixed effects | Estimate  (95% CI) | Std. Error | DF | t-value | p-value |
| --- | --- | --- | --- | --- | --- |
| Intercept | 4.08  (3.64, 4.52) | 0.22 | 1631 | 18.4 | 0.0000 |
| Effort (log transformed) | 1.14  (0.99, 1.30) | 0.08 | 1631 | 14.5 | 0.0000 |
| Longitude (centered, smoothed) | -0.48  (-1.31, 0.35) | 0.42 | 1631 | -1.14 | 0.25 |
|  |  |  |  |  |  |
| Random effects | Intercept | Residual |  |  |  |
| Year | 0.33 | 0.84 |  |  |  |

Results from an additive mixed effect regression with log-transformed abundance as the response term, trapping effort as an intercept offset, trapping location (latitude, centered) as a smoothing term, and year as a random effect

| Fixed effects | Estimate  (95% CI) | Std. Error | DF | t-value | p-value |
| --- | --- | --- | --- | --- | --- |
| Intercept | 4.34  (3.88, 4.79) | 0.23 | 1630 | 18.7 | 0.0000 |
| Effort (log transformed) | 1.05  (0.88, 1.21) | 0.08 | 1630 | 12.7 | 0.0000 |
| s(cLong, sLat) Fx1 | 0.24  (-0.007, 0.44) | 0.11 | 1630 | 1.88 | 0.06 |
| s(cLong, sLat) Fx2 | 0.05  (-0.17, 0.26) | 0.11 | 1630 | 0.42 | 0.67 |
|  |  |  |  |  |  |
| Random effects | Intercept | Residual |  |  |  |
| Year | 0.34 | 0.78 |  |  |  |

Results from an additive mixed effect regression with log-transformed abundance as the response term, trapping effort as fixed effect, trapping location (longitude by latitude, centered) as a smoothing term, and year as a random effect.

GLMM and GAMM regressions of annual site-level mosquito species richness by year

| Fixed effects | Estimate (95% CI) | Std. Error | t-value |
| --- | --- | --- | --- |
| Intercept | -2.63 (-3.08, -2.18) | 0.23 | -11.5 |
| Year (centered) | 0.1 (0.08, 0.11) | 0.01 | 9.46 |
|  |  |  |  |
| Random effects | Variance | Std. Dev |  |
| Site | 4.26 | 2.07 |  |
| Residual | 5.00 | 2.24 |  |

Results from a mixed effect linear regression of centered-mosquito species richness in light traps set at 87 trapping locations in Connecticut, U.S. from 2001 – 2019.

| Fixed effects | Estimate  (95% CI) | Std. Error | DF | t-value | p-value |
| --- | --- | --- | --- | --- | --- |
| Intercept | -6.52  (-7.85, -5.18) | 0.68 | 1563 | -9.58 | 0.0000 |
| Effort (log transformed) | 2.48  (2.00, 2.95) | 0.24 | 1563 | 10.2 | 0.0000 |
| Year (centered, smoothed) | 1.78  (-0.03, 3.6) | 0.93 | 1563 | 193 | 0.05 |
|  |  |  |  |  |  |
| Random effects | Intercept | Residual |  |  |  |
| Site | 2.08 | 2.15 |  |  |  |

Results from a mixed effect additive regression of centered-mosquito species richness in light traps set at 87 trapping locations in Connecticut, U.S. from 2001 – 2019.

GLMM regressions of total site-level species richness by latitude and longitude

| Fixed effects | Estimate (95% CI) | Std. Error | t-value |
| --- | --- | --- | --- |
| Intercept | -2.63  (-3.18, -2.08) | 0.27 | -9.91 |
| Latitude (centered) | 0.63  (0.05, 1.21) | 0.30 | 2.11 |
|  |  |  |  |
| Random effects | Variance | Std. Dev |  |
| Year | 1.24 | 1.12 |  |
| Residual | 8.26 | 2.88 |  |

Results from a generalized mixed effect regression with centered species richness as the response term, trapping effort as an intercept offset, trapping location (latitude, centered) as a predictor term, and year as a random effect.

| Fixed effects | Estimate (95% CI) | Std. Error | z-value |
| --- | --- | --- | --- |
| Intercept | -2.63  (-3.18, -2.08) | 0.27 | -9.91 |
| Longitude (centered) | 1.26  (0.99, 1.52) | 0.13 | 9.34 |
|  |  |  |  |
| Random effects | Variance | Std. Dev |  |
| Year | 1.25 | 1.12 |  |
| Residual | 7.87 | 2.80 |  |

Results from a generalized mixed effect regression with centered species richness as the response term, trapping effort as an intercept offset, trapping location (latitude, centered) as a predictor term, and year as a random effect.

GAMM regressions of total site-level species richness by latitude and longitude

| Fixed effects | Estimate  (95% CI) | Std. Error | DF | t-value | p-value |
| --- | --- | --- | --- | --- | --- |
| Intercept | -6.42  (-7.89, -4.96) | 0.75 | 1631 | -8.60 | 0.0000 |
| Effort (log transformed) | 2.44  (1.92, 2.96) | 0.27 | 1631 | 9.14 | 0.0000 |
| Latitude (centered, smoothed) | -0.52  (-2.34, 1.29) | 0.93 | 1631 | -0.56 | 0.57 |
|  |  |  |  |  |  |
| Random effects | Intercept | Residual |  |  |  |
| Year | 1.06 | 2.77 |  |  |  |

Results from an additive mixed effect regression with species richness as the response term, trapping effort as an intercept offset, trapping location (latitude, centered) as a smoothing term, and year as a random effect

| Fixed effects | Estimate  (95% CI) | Std. Error | DF | t-value | p-value |
| --- | --- | --- | --- | --- | --- |
| Intercept | -4.88  (-6.29, -3.48) | 0.72 | 1631 | -6.80 | 0.0000 |
| Effort (log transformed) | 1.85  (1.36, 2.35) | 0.25 | 1631 | 7.28 | 0.0000 |
| Longitude (smoothed) | -4.56  (-7.09, -2.20) | 1.29 | 1631 | -3.54 | 0.0004 |
|  |  |  |  |  |  |
| Random effects | Intercept | Residual |  |  |  |
| Year | 1.09 | 2.72 |  |  |  |

Results from an additive mixed effect regression with species richness as the response term, trapping effort as an intercept offset, trapping location (longitude, centered) as a smoothing term, and year as a random effect

| Fixed effects | Estimate  (95% CI) | Std. Error | DF | t-value | p-value |
| --- | --- | --- | --- | --- | --- |
| Intercept | -6.35  (-7.78, -4.92) | 0.73 | 1630 | -8.68 | 0.0000 |
| Effort (log transformed) | 2.42  (1.90, 2.93) | 0.26 | 1630 | 9.26 | 0.0000 |
| s(cLong, sLat) Fx1 | 1.45  (0.74, 2.17) | 0.37 | 1630 | 3.97 | 0.0001 |
| s(cLong, sLat) Fx2 | 0.70  (0.01, 1.39) | 0.35 | 1630 | 1.99 | 0.05 |
|  |  |  |  |  |  |
| Random effects | Intercept | Residual |  |  |  |
| Year | 1.07 | 2.46 |  |  |  |

Results from an additive mixed effect regression with centered richness as the response term, trapping effort as fixed effect, trapping location (longitude by latitude, centered) as a smoothing term, and year as a random effect.

GLMM and GAMM regressions of annual site-level mosquito species evenness by year

| Fixed effects | Estimate (95% CI) | Std. Error | t-value |
| --- | --- | --- | --- |
| Intercept | -2.63  (-2.69, -2.57) | 0.03 | -83.1 |
| Year | -0.01  (-0.02, -0.01) | 0.002 | -7.86 |
|  |  |  |  |
| Random effects | Variance | Std. Dev |  |
| Site | 0.08 | 0.28 |  |
| Residual | 0.14 | 0.38 |  |

Results from a mixed effect linear regression of centered-mosquito species evenness in light traps in Connecticut, U.S. from 2001 – 2019.

| Fixed effects | Estimate  (95% CI) | Std. Error | DF | t-value | p-value |
| --- | --- | --- | --- | --- | --- |
| Intercept | -0.12  (-0.30, 0.06) | 0.09 | 1563 | -1.30 | 0.19 |
| Effort (log transformed) | 0.05  (-0.02, 0.11) | 0.03 | 1563 | 1.35 | 0.18 |
| Year (smoothed) | -0.04  (-0.06, -0.03) | 0.008 | 1563 | -5.58 | 0.0000 |
|  |  |  |  |  |  |
| Random effects | Intercept | Residual |  |  |  |
| Site | 0.22 | 0.31 |  |  |  |

Results from a mixed effect additive regression of centered-mosquito species evenness in light traps in Connecticut, U.S. from 2001 – 2019.

GLMM regressions of total site-level species evenness by latitude and longitude

| Fixed effects | Estimate (95% CI) | Std. Error | t-value |
| --- | --- | --- | --- |
| Intercept | -2.63  (-2.69, -2.57) | 0.03 | -90.75 |
| Latitude (centered) | 0.36  (0.27, 0.45) | 0.05 | 7.63 |
|  |  |  |  |
| Random effects | Variance | Std. Dev |  |
| Year | 0.01 | 0.12 |  |
| Residual | 0.21 | 0.45 |  |

Results from a generalized mixed effect regression with species evenness as the response term, trapping effort as an intercept offset, trapping location (latitude, centered) as a predictor term, and year as a random effect.

| Fixed effects | Estimate (95% CI) | Std. Error | t-value |
| --- | --- | --- | --- |
| Intercept | -2.63  (-2.69, -2.57) | 0.03 | -90.8 |
| Longitude (centered) | 0.18  (0.14, 0.23) | 0.02 | 8.54 |
|  |  |  |  |
| Random effects | Variance | Std. Dev |  |
| Year | 0.01 | 0.12 |  |
| Residual | 0.21 | 0.45 |  |

Results from a generalized mixed effect regression with species evenness as the response term, trapping effort as an intercept offset, trapping location (latitude, centered) as a predictor term, and year as a random effect.

GAMM regressions of total site-level species evenness by latitude and longitude

| Fixed effects | Estimate  (95% CI) | Std. Error | DF | t-value | p-value |
| --- | --- | --- | --- | --- | --- |
| Intercept | -0.06  (-0.25, 0.12) | 0.10 | 1631 | -0.66 | 0.51 |
| Effort (log transformed) | 0.02  (-0.05, 0.10) | 0.04 | 1631 | 0.68 | 0.50 |
| Latitude  (smoothed) | -0.13  (-0.35, 0.08) | 0.11 | 1631 | -1.21 | 0.24 |
|  |  |  |  |  |  |
| Random effects | Intercept | Residual |  |  |  |
| Year | 0.09 | 0.37 |  |  |  |

Results from an additive mixed effect regression with species evenness as the response term, trapping effort as an intercept offset, trapping location (latitude, centered) as a smoothing term, and year as a random effect

| Fixed effects | Estimate  (95% CI) | Std. Error | DF | t-value | p-value |
| --- | --- | --- | --- | --- | --- |
| Intercept | 0.04  (-0.14, 0.22) | 0.09 | 1631 | 0.44 | 0.66 |
| Effort (log transformed) | -0.02  (-0.08, 0.05) | 0.03 | 1631 | -0.45 | 0.65 |
| Longitude  (smoothed) | -0.43  (-0.74, -0.11) | 0.16 | 1631 | -2.64 | 0.008 |
|  |  |  |  |  |  |
| Random effects | Intercept | Residual |  |  |  |
| Year | 0.09 | 0.36 |  |  |  |

Results from an additive mixed effect regression with species evenness as the response term, trapping effort as an intercept offset, trapping location (Longitude, centered) as a smoothing term, and year as a random effect

| Fixed effects | Estimate  (95% CI) | Std. Error | DF | t-value | p-value |
| --- | --- | --- | --- | --- | --- |
| Intercept | -0.02  (-0.22, 0.17) | 0.10 | 1630 | -0.22 | 0.83 |
| Effort (log transformed) | 0.008  (-0.06, 0.08) | 0.04 | 1630 | 0.22 | 0.82 |
| s(cLong, sLat) Fx1 | 0.12  (0.02, 0.21) | 0.05 | 1630 | 2.47 | 0.01 |
| s(cLong, sLat) Fx2 | 0.16  (0.04, 0.22) | 0.04 | 1630 | 2.93 | 0.004 |
|  |  |  |  |  |  |
| Random effects | Intercept | Residual |  |  |  |
| Year | 0.09 | 0.35 |  |  |  |

Results from an additive mixed effect regression with centered evenness as the response term, trapping effort as fixed effect, trapping location (longitude by latitude, centered) as a smoothing term, and year as a random effect.

GLMM and GAMM regressions of the annual prevalence of single mosquito species detections among sites by year

| Fixed effects | Estimate (95% CI) | Std. Error | t-value | Pr(>\|z\|) |
| --- | --- | --- | --- | --- |
| Intercept | -8.90 (-10.5, -7.93) | 0.61 | -14.7 | <2e-16 |
| Year  (centered) | -0.13 (-0.37, 0.06) | 0.01 | -1.22 | 0.22 |
|  |  |  |  |  |
| Random effects | Variance | Std. Dev |  |  |
| Site | 0 | 0 |  |  |

Results from a binomial-error linear mixed effects regression of the proportion of single mosquito species collections in light traps set at 87 trapping locations in Connecticut, U.S. from 2001 – 2019.

| Fixed effects | Estimate  (95% CI) | Std. Error | DF | t-value | p-value |
| --- | --- | --- | --- | --- | --- |
| Intercept | 0.009 (-1.16, 1.18) | 0.60 | 1563 | 0.02 | 0.99 |
| Effort (log transformed) | -0.50 (-0.95, -0.06) | 0.23 | 1563 | -2.21 | 0.03 |
| Year (centered, smoothed) | -0.02 (-0.14, 0.10) | 0.06 | 1563 | -0.31 | 0.75 |
|  |  |  |  |  |  |
| Random effects | Intercept | Residual |  |  |  |
| Site | 0.46e-5 | 1 |  |  |  |

Results from an additive binomial-error regression of the proportion of single mosquito species collections in light traps set at 87 trapping locations in Connecticut, U.S. from 2001 – 2019.

GLMM regressions of the annual prevalence of single mosquito species detections among sites by latitude and longitude

| Fixed effects | Estimate (95% CI) | Std. Error | t-value | Pr(>\|z\|) |
| --- | --- | --- | --- | --- |
| Intercept | -11.7 (-17.2, -9.02) | 1.98 | -5.93 | 2.98e-9 |
| Latitude  (centered) | 10.8 (4.60, 21.2) | 4.1 | 2.66 | 0.008 |
|  |  |  |  |  |
| Random effects | Variance | Std. Dev |  |  |
| Year | 0 | 0 |  |  |

Results from a binomial-error linear mixed effects regression of the proportion of single mosquito species collections in light traps set at 87 trapping locations in Connecticut, U.S. from 2001 – 2019.

| Fixed effects | Estimate (95% CI) | Std. Error | t-value | Pr(>\|z\|) |
| --- | --- | --- | --- | --- |
| Intercept | -8.73 (-9.97, -7.87) | 0.52 | -16.9 | <2e-16 |
| Longitude  (centered) | 0.56 (-1.38, 2.36) | 0.91 | 0.62 | 0.54 |
|  |  |  |  |  |
| Random effects | Variance | Std. Dev |  |  |
| Year | 0 | 0 |  |  |

Results from a binomial-error linear mixed effects regression of the proportion of single mosquito species collections in light traps set at 87 trapping locations in Connecticut, U.S. from 2001 – 2019.

GAMM regressions of the annual prevalence of single mosquito species detections among sites by latitude and longitude

| Fixed effects | Estimate  (95% CI) | Std. Error | DF | t-value | p-value |
| --- | --- | --- | --- | --- | --- |
| Intercept | 0.14 (-1.05, 1.33) | 0.61 | 1631 | 0.23 | 0.82 |
| Effort (log transformed) | -0.55 (-1.01, -0.10) | 0.23 | 1631 | -2.39 | 0.02 |
| Latitude (centered, smoothed) | -0.05 (-0.17, 0.07) | 0.06 | 1631 | -0.83 | 0.41 |
|  |  |  |  |  |  |
| Random effects | Intercept | Residual |  |  |  |
| Year | 3.74e-5 | 1 |  |  |  |

Results from an additive binomial-error regression of the proportion of single mosquito species collections in light traps set at 87 trapping locations in Connecticut, U.S. from 2001 – 2019.

| Fixed effects | Estimate  (95% CI) | Std. Error | DF | t-value | p-value |
| --- | --- | --- | --- | --- | --- |
| Intercept | 0.06 (-1.10, 1.23) | 0.60 | 1631 | 0.11 | 0.91 |
| Effort (log transformed) | -0.52 (-0.97, -0.08) | 0.23 | 1631 | -2.32 | 0.02 |
| Longitude (centered, smoothed) | -0.07 (-0.19, 0.05) | 0.06 | 1631 | -1.10 | 0.28 |
|  |  |  |  |  |  |
| Random effects | Intercept | Residual |  |  |  |
| Year | 4.03e-6 | 1 |  |  |  |

Results from an additive binomial-error regression of the proportion of single mosquito species collections in light traps set at 87 trapping locations in Connecticut, U.S. from 2001 – 2019.

| Fixed effects | Estimate  (95% CI) | Std. Error | DF | t-value | p-value |
| --- | --- | --- | --- | --- | --- |
| Intercept | -0.11  (-1.08, 1.30) | 0.61 | 1630 | 0.18 | 0.86 |
| Effort (log transformed) | -0.54  (-0.99, -0.09) | 0.23 | 1630 | -2.33 | 0.02 |
| s(cLong, sLat) Fx1 | 0.02  (-0.15, 0.19) | 0.09 | 1630 | 0.22 | 0.83 |
| s(cLong, sLat) Fx2 | 0.06  (-0.04, 0.15) | 0.05 | 1630 | 1.13 | 0.26 |
|  |  |  |  |  |  |
| Random effects | Intercept | Residual |  |  |  |
| Year | 4.5e-5 | 1 |  |  |  |

Results from an additive mixed effect regression with proportion of single mosquito species collections, trapping effort as fixed effect, trapping location (longitude by latitude, centered) as a smoothing term, and year as a random effect.

GLMM and GAMM regressions of total species-level collections by year

| Fixed effects | Estimate (95% CI) | Std. Error | t-value |
| --- | --- | --- | --- |
| Intercept | -105.5  (-132.3, -78.7) | 13.7 | -7.73 |
| Year | 0.051 (0.04, 0.06) | 0.007 | 7.59 |
|  |  |  |  |
| Random effects | Variance | Std. Dev |  |
| Species | 11.9 | 3.45 |  |
| Residual | 1.21 | 1.10 |  |

Results from a mixed effect linear regression of the log-transformed annual collection of mosquito species collected in light traps in Connecticut, U.S. from 2001 – 2019.

| Fixed effects | Estimate  (95% CI) | Std. Error | DF | t-value | p-value |
| --- | --- | --- | --- | --- | --- |
| Intercept | -2.76 (-9.96, 4.43) | 3.67 | 826 | -0.75 | 0.45 |
| Effort (log transformed) | 1.10 (0.10, 2.10) | 0.51 | 826 | 2.15 | 0.03 |
| Year (smoothed) | 0.33 (0.10, 0.55) | 0.12 | 826 | 2.85 | 0.0045 |
|  |  |  |  |  |  |
| Random effects | Intercept | Residual |  |  |  |
| Species | 3.41 | 1.10 |  |  |  |

Results from a mixed effect additive regression of the log-transformed annual collection of mosquito species collected in light traps in Connecticut, U.S. from 2001 – 2019.

GLMM and GAMM regressions of the prevalence of single-site detections among species by year

| Fixed effects | Estimate (95% CI) | Std. Error | t-value |
| --- | --- | --- | --- |
| Intercept | -7.05 (-7.14, -6.96) | 0.05 | -153.8 |
| Year (centered) | -0.01  (-0.013, -0.008) | 0.001 | -8.47 |
|  |  |  |  |
| Random effects | Variance | Std. Dev |  |
| Species | 0.10 | 0.31 |  |
| Residual | 0.03 | 0.18 |  |

Results from a mixed effect linear regression of the proportion of single-week collections of mosquito species in light traps in Connecticut, U.S. from 2001 – 2019.

| Fixed effects | Estimate  (95% CI) | Std. Error | DF | t-value | p-value |
| --- | --- | --- | --- | --- | --- |
| Intercept | 0.99 (-0.10, 2.08) | 0.56 | 647 | 1.78 | 0.08 |
| Effort (log transformed) | -0.13 (-0.28, 0.03) | 0.08 | 647 | -1.63 | 0.1 |
| Year (smoothed) | -0.02 (-0.04, -0.009) | 0.007 | 647 | -3.35 | 0.0009 |
|  |  |  |  |  |  |
| Random effects | Intercept | Residual |  |  |  |
| Species | 0.31 | 0.16 |  |  |  |

Results from a mixed effect additive binomial-error regression of the proportion of single-week collections of mosquito species in light traps in Connecticut, U.S. from 2001 – 2019.

| Species | Annual Collections | | Annual Detections | |
| --- | --- | --- | --- | --- |
|  | Estimate | p-value | Estimate | p-value |
| *Aedes abserratus* | 0.03 | 0.23 | 0.008 | 0.20 |
| ***Aedes***  ***albopictus*** | ***0.52*** | ***0.004*** | ***0.23*** | ***8.6e-10*** |
| *Aedes atlanticus* | 0.27 | 0.34 | 0.23 | 0.10 |
| *Aedes atropalpus* | -0.08 | 0.14 | -0.06 | 0.38 |
| *Aedes aurifer* | 0.05 | 0.15 | ***0.02*** | ***0.02*** |
| *Aedes canadensis* | 0.03 | 0.17 | -0.006 | 0.19 |
| *Aedes cantator* | -0.01 | 0.55 | ***-0.03*** | ***5.9e-8*** |
| *Aedes cinereus* | -0.02 | 0.38 | ***-0.01*** | ***0.02*** |
| *Aedes communis* | -0.09 | 0.13 | ***-0.05*** | ***0.04*** |
| *Aedes diantaeus* | -0.05 | 0.41 | -0.001 | 0.99 |
| *Aedes excrucians* | -0.009 | 0.80 | ***-0.01*** | ***0.03*** |
| *Aedes fitchii* | 0.12 | 0.14 | ***0.1*** | ***0.02*** |
| *Aedes grossbecki* | 0.15 | 0.06 | ***0.11*** | ***0.001*** |
| *Aedes hendersoni* | -0.002 | 0.39 | -0.002 | 0.98 |
| *Aedes infirmatus* | NA | NA | NA | NA |
| *Aedes intrudens* | 0.32 | 0.10 | 0.07 | 0.72 |
| *Aedes japonicus* | ***0.05*** | ***0.002*** | -0.0001 | 0.98 |
| *Aedes provocans* | 0.13 | 0.08 | ***0.05*** | ***0.03*** |
| *Aedes sollicitans* | ***-0.08*** | ***0.02*** | -0.02 | 0.07 |
| ***Aedes***  ***sticticus*** | ***-0.16*** | ***0.02*** | ***-0.05*** | ***< 2e-16*** |
| *Aedes stimulans* | -0.03 | 0.33 | ***-0.03*** | ***1.5e-6*** |
| ***Aedes taeniorhynchus*** | ***0.05*** | ***0.01*** | ***0.02*** | ***0.03*** |
| *Aedes thibaulti* | 0.04 | 0.20 | 0.01 | 0.13 |
| *Aedes triseriatus* | ***-0.05*** | ***0.02*** | -0.006 | 0.21 |
| ***Aedes***  ***trivittatus*** | ***-0.11*** | ***0.04*** | ***-0.02*** | ***0.002*** |
| *Aedes vexans* | -0.02 | 0.45 | -0.007 | 0.11 |
| *Anopheles barberi* | -0.002 | 0.97 | -0.02 | 0.66 |
| ***Anopheles crucians*** | ***0.18*** | ***0.05*** | ***0.11*** | ***7.6e-12*** |
| *Anopheles punctipennis* | ***0.04*** | ***0.04*** | -0.004 | 0.33 |
| ***Anopheles quadrimaculatus*** | ***0.06*** | ***0.0008*** | ***0.01*** | ***0.008*** |
| ***Anopheles walkeri*** | ***0.10*** | ***0.0007*** | ***0.03*** | ***0.0001*** |
| *Coquilettedia perturbans* | ***0.05*** | ***0.005*** | -0.004 | 0.37 |
| *Culiseta melanura* | 0.03 | 0.27 | -0.008 | 0.10 |
| *Culiseta minnesotae* | -0.02 | 0.67 | 0.002 | 0.91 |
| *Culiseta morsitans* | -0.03 | 0.55 | ***-0.02*** | ***0.03*** |
| ***Culex***  ***erraticus*** | ***0.47*** | ***0.02*** | ***0.27*** | ***9.5e-10*** |
| *Culex pipiens* | -0.03 | 0.11 | ***-0.01*** | ***0.01*** |
| *Culex restuans* | -0.003 | 0.89 | -0.004 | 0.44 |
| *Culex salinarius* | ***0.08*** | ***0.005*** | 0.003 | 0.52 |
| ***Culex***  ***territans*** | ***0.06*** | ***0.04*** | ***0.03*** | ***8.2e-6*** |
| *Orthopodemia signifera* | 0.04 | 0.27 | 0.04 | 0.24 |
| *Psorophora ciliate* | -0.51 | 0.40 | -0.26 | 0.30 |
| ***Psorophora columbiae*** | ***0.26*** | ***0.02*** | ***0.18*** | ***0.001*** |
| *Psorophora ferox* | 0.04 | 0.39 | 0.002 | 0.63 |
| ***Psorophora howardii*** | ***0.33*** | ***0.0001*** | ***0.27*** | ***5.45e-8*** |
| *Uranotenia sapphirina* | 0.004 | 0.73 | -0.002 | 0.67 |

Results from linear regressions of the total annual collection (log-transformed) OR number of sites detected by year with an offset for annual trapping effort: regressions of sites detections used a Poisson-error distribution. Mosquitoes were collected in light traps from 87 standard surveillance sites in Connecticut, U.S. from 2001 – 2019.
